# Historical Epidemics Cartography Generated by Spatial Analysis: Mapping the Heterogeneity of Three Medieval "Plagues" in Dijon

**DOI:** 10.1371/journal.pone.0143866

**Published:** 2015-12-01

**Authors:** Pierre Galanaud, Anne Galanaud, Patrick Giraudoux

**Affiliations:** 1 UMR996, Inflammation, Chemokines and Immunopathology, Inserm, Univ Paris-Sud, Université Paris-Saclay, 92140, Clamart, France; 2 75014, Paris, France; 3 Chrono-environnement, Université de Bourgogne-Franche-Comté, CNRS UMR6249, Besançon, France & Institut Universitaire de France, Paris, France; Columbia University, UNITED STATES

## Abstract

**Objectives:**

This work was designed to adapt Geographical Information System-based spatial analysis to the study of historical epidemics. We mapped "plague" deaths during three epidemics of the early 15th century, analyzed spatial distributions by applying the Kulldorff's method, and determined their relationships with the distribution of socio-professional categories in the city of Dijon.

**Materials and Methods:**

Our study was based on a database including 50 annual tax registers (established from 1376 to 1447) indicating deaths and survivors among the heads of households, their home location, tax level and profession. The households of the deceased and survivors during 6 years with excess mortality were individually located on a georeferenced medieval map, established by taking advantage of the preserved geography of the historical center of Dijon. We searched for clusters of heads of households characterized by shared tax levels (high-tax payers, the upper decile; low-tax payers, the half charged at the minimum level) or professional activities and for clusters of differential mortality.

**Results:**

High-tax payers were preferentially in the northern intramural part, as well as most wealthy or specialized professionals, whereas low-tax payers were preferentially in the southern part. During two epidemics, in 1400–1401 and 1428, areas of higher mortality were found in the northern part whereas areas of lower mortality were in the southern one. A high concentration of housing and the proximity to food stocks were common features of the most affected areas, creating suitable conditions for rats to pullulate. A third epidemic, lasting from 1438 to 1440 had a different and evolving geography: cases were initially concentrated around the southern gate, at the confluence of three rivers, they were then diffuse, and ended with residual foci of deaths in the northern suburb.

**Conclusion:**

Using a selected historical source, we designed an approach allowing spatial analysis of urban medieval epidemics. Our results fit with the view that the 1400–1401 epidemic was a Black Death recurrence. They suggest that this was also the case in 1428, whereas in 1438–1440 a different, possibly waterborne, disease was involved.

## Introduction

The study of past diseases, aside from being of historical and demographic interest, may contribute to a better understanding of the spread of epidemics in the absence of medical treatment. Whereas the epidemiology of todays' communicable diseases has benefited from the application of Geographical Information System (GIS)-based spatial analysis [1, 2], the cartographic representation and statistical analysis of ancient epidemics has been limited. The geography of deaths has been delineated for a small number of historical epidemics, such as the 17th century Great Plague of London [3, 4], the epidemics that affected London from the mid-16th century to the end of the 17th century [5] or the 1430 plague in Florence [6]. However, although a global spatiotemporal modeling of medieval Black Death (14th century Western Europe) in comparison with bubonic plague (19–20th century Indian subcontinent) outlined the feasibility of cartography based on selected historical databases [7] the fine grain cartographic representation of medieval epidemics has not, so far as we know, benefitted from the application of GIS-based spatial analysis. We undertook a cartographic study of three successive medieval epidemics that appeared suitable for the adaptation of a GIS-based spatial analysis approach.

We selected epidemics that struck the city of Dijon during the first half of the 15th century. This choice was based on the availability of informative historical sources and on the potential for adequate cartographic reconstitution. Our study is mainly based on the annual registers of the *marcs* tax, which provide information on the demography of heads of households and on the trend of deaths in late medieval Dijon [8, 9]. These documents are unique because of their continuity over time and because they indicate the location of households, an exceptional feature in contemporary documents. The cartographic reconstruction took advantage of the preservation of the historical centre of Dijon, and of the numerous sources and published studies that shed light on the concordance between the medieval and modern streets. This allowed us to locate the streets cited by the 15th century tax collector to draw a georeferenced medieval map that we used for the tentative individual assignment of heads of households. On this basis, we mapped "plague" deaths, analyzed spatial distributions and determined their relationships with the distribution of socio-professional categories in the city.

The Black Death, the mid-14th century plague that led to a major depopulation in Asia and Europe, returned periodically to Western Europe for several centuries, arising in distinct places with variable geographic extension and intensity [10, 11]. Although it should be kept in mind that not every deadly epidemic disease that affected medieval populations corresponded with a new outbreak of plague [6], the medieval and early modern disease exhibits a changing pattern, as outlined by the diversity of plague recurrences in London from the 14th to the 17th century [5, 12]. The mechanisms underlying this changing pattern as well as the differences between the 14th century deadly epidemic and the modern outbreaks of bubonic plague [7] are not fully understood. The molecular identification of *Yersinia pestis* as the causative agent of the Black Death and a number of later outbreaks of plague [13, 14, 15], aside from representing a major advance in the history of communicable diseases, allowed comparison of the Black Death bacterial genome against modern genomes [16, 17] in order to understand the organism's evolution. Although genetic changes of the bacteria may be partially responsible for differences in disease manifestations and severity, other factors such as environment, vector dynamics and host susceptibility should be taken into account [16]. A number of these factors, such as urban characteristics, can be analyzed by the application of modern statistical tools to selected historical sources.

In Dijon, the Black Death is reflected in historical sources a decade later, in the first preserved *marcs* tax registers, by the dramatic excess of taxpayers additionally charged for widowed spouses or as guardians of orphans [18]. The first widespread recurrence of plague in 1360–1363 and the milder epidemic of 1374 [10] occurred in years when the *marcs* tax registers are not preserved. In the first half of the 15th century, three epidemics, either referred as widespread recurrences of the Black Death [10] or documented in local historical sources [19], were associated with a detectable excess mortality in the Dijon *marcs* tax registers: 1400–1401, 1428 and 1438–1440. These six "years of plague" were therefore selected for study.

At that time, Dijon benefited from its position within a "Burgundian State" that included in addition to Burgundy itself the present *Franche-Comté* and a large part of present Belgium and the Netherlands. The city, at the junction of roads connecting Paris, Flanders, Italy and the papal city of Avignon, had approximately 10,000 inhabitants when Ghent (included in the "Burgundian State") and Paris respectively counted 60,000 and 80,000 inhabitants. Its socio-economic and professional geography was characterized by a division between the northern intramural component more involved in trade and crafts, and the southern part, in which the population was less dense and religious activities predominated [20].

## Materials and Methods

### Historical sources and database

The main historical source is the *marcs* tax registers of Dijon. These registers annually recorded heads of households in Dijon, indicating their name, amount of tax or lack of tax for exemption, death or departure, their home location (parish and street) and in some cases their profession [8, 9]. Additional information such as a move within or outside the city, or an event likely to alter the tax level (marriage, death of the spouse) may be included **S1 Text**.

With few exceptions over the long duration, clerks initiated the annual registration of heads of households from the same place and followed a virtually identical path that was recorded into the registers. Parishes and street names were listed at the top of the relevant list and changes in direction or street side were often indicated, as well as a number of landmarks, some of which are still preserved.

The database was previously established by one of us (AG) from this unique historical source [21]. It is based on an adjustment of the database management application *Quatrième Dimension*, specially designed for the study, and includes 50 annual registers established from 1376 to 1447 **S1 Text**.

This allowed us to define tentative individuals characterized by reference to (and position within) the page of a register, standardized name, home location, socio-economic status (amount of tax or tax exemption and its grounds), profession (when indicated or found in other source materials), duration of listing in the registers and mode of disappearance from them.

### Demographic information from the registers

#### Registration of the heads of households

Each annual register appears as the updating of the previous one, with corrections for the addition of newly registered heads of household or the indication of those who are no more present, because of death, of leave from the city or of a move within the city (in this case the clerk indicates where to find the new home location). The updating was performed between February and March. Thus, those deaths indicated in a given register approximately corresponded to the cumulative deaths of the previous year, while the survivors were those present on the first months of the year. The demography of each "year of plague" is thus reflected in the register established at the beginning of the following year. As an example, the plague of 1400–1401 is analyzed from the 1401–1402 registers. In a limited number of cases, information on the approximate time of death could be obtained, either because the register provided such an indication for a number of the deceased (this occurred essentially for the deaths of year 1401) or because we had access to inventories of inheritance of a sizeable number of deceased (this was the case for the deaths of 1438–1440 [21]).

The living heads of households taken into account to number the survivors were those reported as present, from whom we excluded a minority of households that did not correspond to individuals **S2 Text**. We assumed that their spatial distribution was representative of that of the population that survived the epidemics, although their number provides an underestimate of the inhabitants at risk of epidemic death **S2 Text**.

#### Selection of "years of plague"

In this work, a "year of plague" is defined by the conjunction of an excess of death in the corresponding *marcs* tax register and a plague report in historical sources.

The excess of death was searched by analysis of the crude mortality rates of heads of households during the years included in the database **S1 Fig**. The mortality rate, which remained below 50 per thousand in most years, displayed two major peaks, in 1400 and in 1439. These years were selected, together with years 1401 and 1438, when mortality rates were unusually high. Year 1440, marked by a marginally increased rate was also taken into account in order to analyze the evolution of the 1438–1439 epidemic which displayed a development more progressive than the brutal 1400 "plague". The year 1428 was selected on the basis of a mortality rate higher than in the previous years (registers for the immediately following years are not available) and because numerous reports of multiple deaths in the same households are highly suggestive of epidemic deaths (see below).

The 1400 and 1439 peaks correspond to widespread epidemic mortality crises [10] and local historical sources document an increased mortality in 1400–1401, 1428 and 1439 **S3 Text**.

#### Counting the deaths

The deaths taken into account were those recorded in the registers, indicated by "dead" or "*defunctus*" and a null tax level, as shown for the year 1400 in S2 Fig, or deduced from the replacement of the head of household by his widow. We assumed that their spatial distribution was representative of that of epidemic mortality. However, the deaths recorded in the registers did not completely correspond with the victims of the epidemic. On the one hand, not every death was due to the epidemic; on the other hand deaths reported by the tax collector were essentially (but not exclusively) those of the heads of households S4 Text. The registers occasionally mentioned the death of another member of the household, provided that its occurrence altered the tax level of the surviving head of household or triggered the allocation of his/her tax to someone else. Of particular interest are reports of concomitant deaths in the same household, as their spatial location might be meaningful during a year of epidemic S4 Text. In this article, this situation was identified as "multiple death" whereas the isolated death of the head of household or of another member of the household was qualified as "single death". Multiple deaths corresponded in most cases to the concomitant death of a head of household and his wife ("dead and his wife") and sometimes to the extinction of an entire nuclear family ("dead wife and children") S4 Text. In addition, a number of death reports closely associated in the registers were considered as grouped in space. They were identified as "grouped deaths" in this article. This was the case of multiple deaths in the same household and of deaths of two contiguous heads of households or of heads of households separated by a single spared household in the same street. The proximity relationships were based on the succession of households in the registers, and those deaths facing in the same street on the map (see below) and separated in the registers were not taken into account, as their positioning was in part arbitrary. We specifically analyzed those deaths of individuals grouped in space, who were more likely to have been victims of the epidemics. Data for the 6 "years of plague" and the previous 3 years are presented in Table 1.

**Table 1:** Demography of the "years of plague" and of the previous years.

Year	1399	**1400**	**1401**	1427	**1428**	1437	**1438**	**1439**	**1440**
Mortality rate	31‰	**175‰**	**51‰**	30‰	**79‰**	22‰	**61‰**	**117‰**	**45‰**
Survivors	1,999	**1,715**	**1,787**	1,869	**1,694**	1,782	**1,684**	**1,522**	**1,740**
Single deaths	66	**322**	**90**	58	**141**	45	**101**	**188**	**68**
Multiple deaths	0	**8**	**4**	0	**95**	2	**14**	**8**	**10**
Total deaths	66	**330**	**94**	58	**236**	47	**115**	**196**	**78**
Death rate	3.2%	**16.1%**	**5%**	3%	**12.2%**	2.6%	**6.4%**	**11.4%**	**4.3%**
Grouped deaths	12	**164**	**20**	2	**140**	2	**37**	**78**	**19**

Legend: Year: the demography of the 6 "years of plague" is in bold characters and the column immediately at the left indicates, for each epidemic, the demography of the previous year. Mortality rate: crude mortality rate (as per thousand) evaluated for the heads of households; reflects the global damage of an epidemic **S1 Fig**. Survivors: number of surviving heads of households after exclusion of those not corresponding to individuals **S2 Text**. Single deaths: number of households with one reported death, whether or not of the head of household. Multiple deaths: number of deaths in the households where the concomitant death of several persons is reported **S4 Text**. Total deaths: total number of deaths taken into account for analysis (sum of lines 4 and 5). Death rate: ratio between the number of reported deaths and the sum of reported deaths and survivors (as percent); does not reflect the mortality of the year, but allows comparisons between groups of individuals or areas during the same year. Grouped deaths: households where multiple deaths took place, or households contiguous or separated by a single survivor in the register.

### Economic information from the registers

#### Tax level

A number of heads of households were exempted, either as members of a group not submitted to the *marcs* tax (nobles, ecclesiastics) or because of an individual exemption, in a minority of cases because of poverty (they were qualified as beggars by the clerk) and in most cases because of a privilege. They will be respectively referred to as exempted for poverty and exempted by privilege S5 Text.

For each taxpayer, the amount of the *marcs* tax stood between an upper limit of 120 sols (2 *marcs*) and a lower limit of 1 sol (1 *marc* is worth 60 sols). About 10% of the taxpayers were charged at or above 20 sols (among whom 20 to 30 paid the maximum amount of 120 sols): they were considered as high-tax payers. About half of the taxpayers were charged at 1 sol: they were considered as low-tax payers **S6 Text**.

Tax level and/or tax status were taken into account for the setting up of socio-economic cartography. Although there are examples of discrepancy between the tax level of an individual and his/her real wealth, the tax level of a group reflects its economic status. The mean tax level of taxpayers within an area was used as an indicator, together with the numbers of high-tax payers (the approximate upper decile), and of low-tax payers (representing the group below the approximate median). The few heads of households exempted for poverty and the heterogeneous group of exempted by privilege were not submitted to statistical analysis, but their numbers were indicated.

As no taxes were levied in the year of death of the deceased, their tax level could be traced from their tax in the register of the previous year, in which they were recorded in most cases. This was used to analyze their relative susceptibility to epidemic mortality, using the heads of households taxed at a different level (or exempted) on the same year as controls.

#### Profession

The indication of a profession was not systematic. Its presence varied from year to year for a given head of household, and increased in frequency over time during the period covered by this work. The professional activity of members of the more prominent families, involved in long distance trading and/or holders of offices were often omitted, although a number of offices (to the city, such as gatekeepers, or to major abbeys or chapels) providing exemption from the *marcs* tax were indicated S1 Table.

### Cartography and georeferencing

The map of medieval Dijon was established by one of us (AG) [21] **S7 Text**. The TIFF scan of the map was georeferenced under Quantum GIS 1.8.0-Lisboa with the corresponding background from the Institut Géographique National SCAN 25® using the Lambert II extended coordinates (EPSG: 27572). This map was used as a base to generate annual vector ESRI shapefiles locating each head of household as a point on his home street, in the sequence used in the registers **S7 Text**.

This positioning in space does not reflect the characteristics of medieval streets, which corresponded to a succession of blocks and dead ends rather than to linear lanes. Although largely arbitrary in the positioning of each individual, the mapping does reflect the closeness between households as expressed by their order in the registers as well as the respective locations of the streets. Our aim was not to reconstitute the occupancy of urban space but to figure out the spatial relationships between the individual heads of households, to search for mortality clusters based on these proximity relationships. We wished to identify any potentially uneven distribution of deaths, taking into account the socio-economic characteristics of the population and the urban spatial organization.

### Statistical analysis

Our goal was to determine whether the deaths (cases) were randomly distributed throughout the city or whether they occurred as epidemic clusters and, that being the case, to localize the clusters and to determine whether the corresponding areas differed from the rest of the urban space in terms of death rate. The same approach was used for the analysis of grouped deaths (in this case, their distribution was analyzed by comparison with the addition of survivors and non-grouped deaths), of tax level categories and of professions.

To this end we used Kulldorff's method [22] implemented in the SaTScan v.9.1.1 software. The null hypothesis is that the observed prevalence follows a binomial distribution. The clusters are considered to be statistically significant if the relative risk values obtained by permutation at random are less than 5 percent higher or equal to those observed in reality. The corresponding areas are considered to be areas of higher or lower mortality relative risk **S8 Text**.

When a statistically significant cluster was detected, the households included in the cluster were selected by the Quantum GIS application and a circular area including the corresponding points was drawn on the georeferenced map. Data for the selected heads of households and their counterparts outside the cluster (controls) were exported from the shapefiles into excel and submitted to statistical analysis. We also confronted the results from mortality-based clusters and tax level-based clusters in order to search for spatial relationships between mortality and socio-economic level distributions.

When a statistically significant cluster happened to approximately correspond to (a) parish(es) the heads of households of the parish(es) were submitted to statistical analysis for comparison with the rest of the city. Four of the Dijon parishes had an extramural component. When appropriate, the parishes that extended on both sides of the rampart (a physical and sociological border) were split into their intra and extramural components.

The qualitative test used for statistical analysis was the chi-square, or the Fischer's exact test in the case of small samples. The quantitative test was the Student's t test.

## Results

### Socio-economic geography of Dijon

#### Spatial analysis of shared tax levels

**Fig 1:**
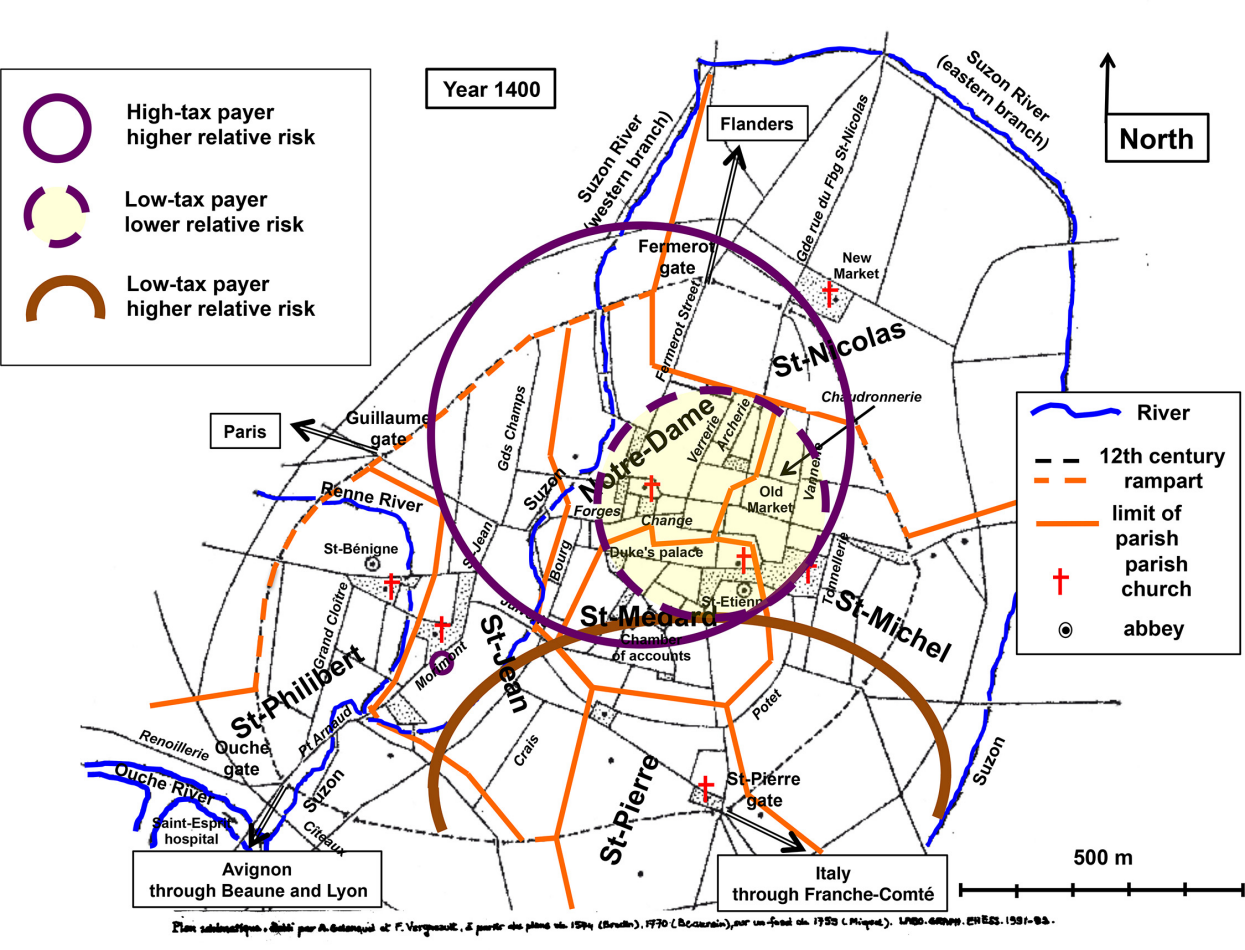
Socio-economic cartography of late medieval Dijon. Georeferenced map of medieval Dijon. Rivers as blue lines. Parish churches, abbeys and a number of prominent places are indicated. Major gates and routes are indicated. The streets mentioned in the text are italicized. The 12th century rampart is shown by dotted lines. Limits of the 7 parishes are in orange solid lines (dotted lines when they coincide with the rampart). Empty areas circled in solid purple line: clusters where high-tax payers were in excess (clusters 1 & 3). Yellow area circled in dotted purple line: cluster where low-tax payers were less numerous (cluster 2). Brown solid line: upper limit of the cluster where low-tax payers were in excess (cluster 4).

Spatial analysis was applied to search for clusters of high-tax payers and of low-tax payers (Fig 1). A major cluster of 771 households with a higher relative risk (RR) for the presence of high-tax payers (RR = 2.83; p < 10^−6^) stood in the northern intramural part of the city (cluster 1). Within this area, a smaller cluster of 242 households where low-tax payers were scarce (RR = 0.49; p < 10^−4^) corresponded to the wealthiest part of Dijon, around the church of *Notre-Dame* and in the commercially active area of the parish of *Saint-Michel*, identified as the *Old Market* (cluster 2). Additionally, five rich neighbours were grouped to constitute a cluster of 5 high-tax payers (RR = 13.16; p = 0.0062) on *Morimont* Street in the vicinity of the wine market (cluster 3). Low-tax payers were in excess in a large cluster of 459 households (RR = 1.50; p < 10^−5^) that occupied most of the south, to the east of *Suzon* River (cluster 4). Cluster 1 and cluster 4 intersected in the central *Saint-Médard* parish, where the political power laid.

Statistical analysis of the tax-level categories within and outside the clusters confirmed that clusters correspond to groups of households with contrasting socio-economic patterns (Table 2). Within cluster 1, the proportion of high-tax payers was, as expected, higher than in the rest of the city, and that of low-tax payers was lower than in the rest of the city. There, the mean tax level of 11.3 sols/taxpayer was higher than in the rest of the city. This pattern was even more pronounced in cluster 2, where the number of high-tax payers nearly equaled that of low-tax payers and the mean tax level amounted 18.1 sols/taxpayer. The 5 heads of households in cluster 3 were taxed between 20 and 120 sols. Cluster 4 offered a contrasting picture with the previous ones with, as expected, a high proportion of low-tax payers whereas high-tax payers were virtually absent. In this area, the mean tax level of 4.3 sols/taxpayer was lower than in the rest of the city.

**Table 2:** Characteristics in the tax level-based clusters.

Cluster:	1	2	3	4	Dijon
Total (N)	771	242	5	459	2,045
High-tax payers (N)	101	45	5	13	160
p (chi-square)	< 10^−11^	< 10^−10^	ND	< 10^−5^	X
Low-tax payers (N)	246	48	0	255	778
p (chi-square)	< 10^−5^	< 10^−9^	ND	< 10^−17^	X
Survivors (N)	644	196	5	399	1,715
Poor exempted (N)	4	1	0	2	15
Exempted by privilege (N)	39	26	0	16	73
Taxpayers (N)	601	169	5	381	1,627
High-tax payers (%)	16.8%	26.6%	100%	3.4%	9.8%
Low-tax payers (%)	40.9%	28.4%	0	66.9%	47.8%
Mean tax (sol/taxpayer)	11.3	18.1	48	4.3	7.4
p (Student's t test)	< 10^−12^	< 10^−18^	ND	< 10^−5^	X

Legend: Line 1: Cluster (or Dijon). Line 2: total number of households (deaths and survivors). Line 3: number of high-tax payers. Line 4: result of the chi-square test comparing the proportions of high-tax payers within and outside the cluster. Line 5: number of low-tax payers. Line 6: result of the chi-square test comparing the proportions of low-tax payers within and outside the cluster. Line 7: number of survivors. Line 8: number of exempted for poverty. Line 9: number of exempted by privilege. Line10: number of taxpayers. Line 11: percent of high-tax payers among the taxpayers. Line 12: percent of low-tax payers among the taxpayers. Line 13: mean tax per taxpayer. Line 14: results of the Student's t test comparing the mean tax level of the cluster to that of the rest of the city. Columns 2–5: data from the clusters. Column 6: data for the whole city. ND: not done. For each cluster, data of the control (rest of the city) can be computed by subtraction from the data of the whole city.

#### Professions in the areas defined by tax level

The geographic distribution of professions differed between tax level-based clusters. Most of the wealthy and/or specialized professionals whose profession was know (S1 Table) were concentrated in the northern intramural part of the city defined by area of the large cluster where high-tax payers were in excess (Fig 1). The majority of merchants, mercers and grocers, who represented the wealthiest professional groups, lived there (33 were settled there, as compared to 8 in the rest of the city; chi-square test, p < 10^−8^), as well as those weavers who were high-tax payers or exempted by privilege and were often prominent drapers (5 out of 7). This area also housed 7 out of 12 barbers or physicians. Virtually all butchers, whose guild was the most powerful of the city, were settled in the *Bourg* district (see below). Most metal craftsmen were also in this area (39 were settled there, as compared to 19 in the rest of the city; chi-square test, p < 0.02) and these were the more specialized, such as the two clock "masters", the sword, knife or spur makers (in *Forges* Street), the coppersmiths (in or around *Chaudronnerie* Street), whereas non specialized blacksmith tended to gather around the gates. In keeping with this pattern oriented towards trade and crafts, the high-tax payers cluster area housed only small numbers of winegrowers (23 as compared to 78 outside the area; chi-square test, p 10^−9^).

### A deadly epidemic in the first years of the century (1400–1401)

#### The 1400–1401 epidemic

A deadly epidemic, considered as the second major recurrence of the Black Death, struck Dijon in 1400, at a time when Burgundy was not directly involved in armed conflicts. The mortality rate of heads of households rose from 31 per thousand in 1399 to 175 per thousand in 1400 before decreasing to 51 per thousand in 1401 (Table 1). In the second year of epidemic, the register indicated the time of death in 35 cases: these deaths occurred at the end of the summer (such as "dead on mid-August") or in the autumn (such as "dead before All Saints' day").

#### Cartography of death

**Fig 2:**
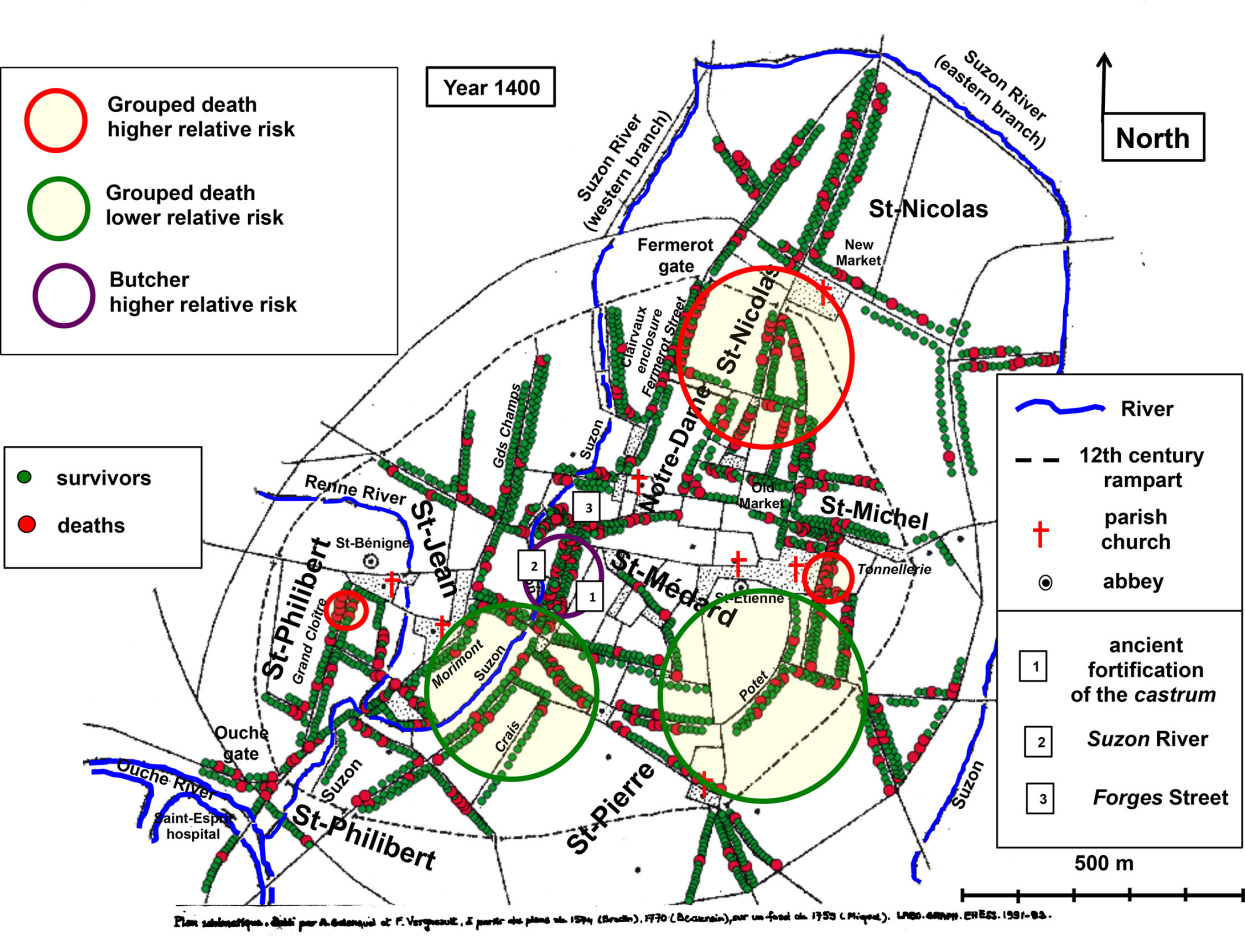
Cartography of death in 1400. Georeferenced map of medieval Dijon. Rivers as blue lines. Parish churches, abbeys and a number of prominent places are indicated. The streets mentioned in the text are italicized. The 12th century rampart is shown by dotted lines. Each point corresponds to a surviving head of household (green points) or to a registered death (larger red points). Yellow areas circled in red: clusters with a higher relative risk of grouped death. Yellow areas circled in green: clusters with a lower or null relative risk of grouped death. Empty area circled in purple: cluster of higher density of butchers in the *Bourg* district. The *Bourg* district stood between the ancient fortification of the *castrum* (1) and the Suzon River (2) and its northern end was contiguous to *Forges* Street (3). Historical evidence of the location of the statistically significant clusters is presented in **S9 Text**.

Spatial analysis of the dead and survivors for 1400 and 1401 did not reveal clusters of higher or lower death relative risk; the instances seemed to be scattered randomly across the city. However, the 164 grouped deaths of 1400 were not distributed at random (Fig 2). Two clusters with a higher relative risk of grouped death were located north of the intramural part of the city. The larger involved 27 grouped deaths among 123 households (RR = 3.08; p = 0.014) and it was at the junction of the *Saint-Nicolas*, *Notre-Dame* and *Saint-Michel* parishes. The smaller involved 8 grouped deaths among 15 households (RR = 6.94; p = 0.040) and was in *Tonnellerie* Street in *Saint-Michel* parish. A third smaller cluster of 5 grouped deaths (RR = 12.83; p = 0.011) was also found on *Grand Cloître* Street in *Saint-Philibert* parish. Two clusters with a lower or null relative risk of grouped death were located in the southern part of the city. One of them was devoid of grouped deaths within 146 households (RR = 0; p = 0.013) and was to the east, centered by *Potet* Street. The other one involved 2 grouped deaths within 190 households (RR = 0.12; p = 0.047) and was to the west, centered by the *Crais* area.

Statistical analysis of the distribution of deaths within and outside the grouped death-based clusters confirmed that they correspond to groups of households with contrasting mortality patterns (Table 3). In the clusters of higher grouped death relative risk, the proportion of deaths (grouped and non-grouped) was higher than in the rest of the city. This was also true when the larger cluster of higher grouped death relative risk was considered alone. In the clusters of lower grouped death relative risk, the proportion of deaths (grouped and non-grouped) was lower than in the rest of the city.

**Table 3:** Deaths in the mortality-based clusters in 1400.

Grouped death clusters	Higher RR (3 clusters)	Higher RR (large cluster)	Lower RR (2 clusters)	Dijon
Total	143	123	336	2,045
Deaths	48	34	40	330
p (chi-square)	< 10^−8^	< 10^−3^	< 0.05	X
Death rate (%)	33.6%	27.6%	11.9%	16.1%

Legend: Line 1: Grouped death clusters (and Dijon). Line 2: total number of households (deaths and survivors). Line 3: number of deaths (grouped and non-grouped). Line 4: result of the chi-square comparing the cluster to the rest of the city. Line 5: death rate as percent. Column 2: pooled data from the 3 clusters of higher grouped death relative risk. Column 3: data restricted to the larger cluster of higher grouped death relative risk (included in the previous column). Column 4: pooled data from the 2 clusters of lower grouped death relative risk. Column 5: data for the whole city. For each cluster, data of the control (rest of the city) can be computed by subtraction from the data of the whole city.

**Fig 3:**
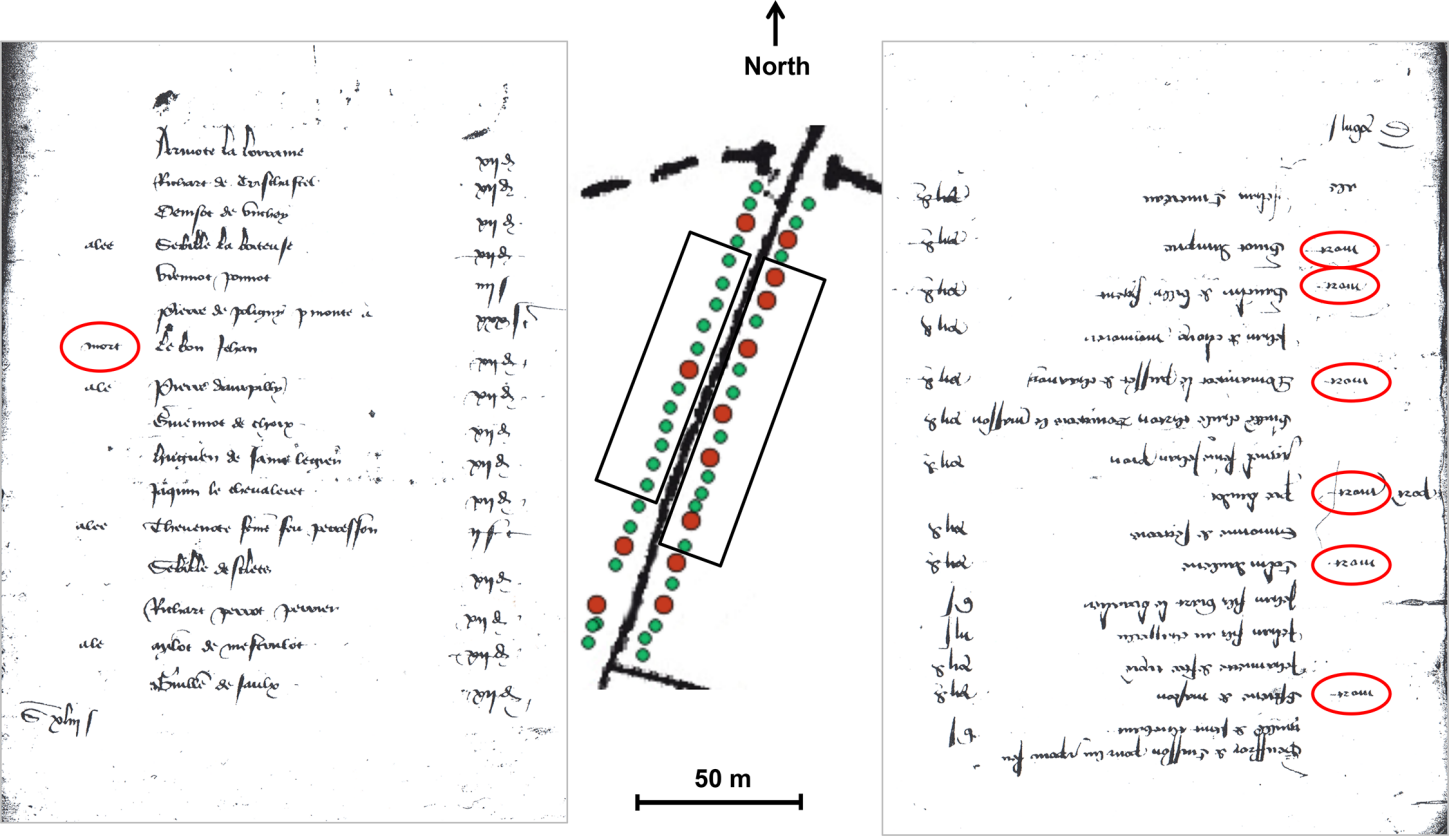
From marcs tax register to cartography of death. A copy of two folios corresponding to either side of *Fermerot* Street in 1400 shows the contrasting mortality between the western and the eastern sides of the street. On the left is folio 1v that enlists households standing on the western side of the street. On the right is folio 2v that enlists households standing on the eastern side of the street. Folio 2v is upside down to fit with the direction of recording (see below). As indicated in the left margin (on the right side for folio 2v), each head of household can be a deceased (*mort*, "dead"), an absent (*ale* or *alee*, "gone") or a survivor (no indication). The red circles indicate the deaths. Deaths are more numerous in folio 2v and all of them correspond to grouped deaths, as they are closely associated with other deaths in the register file (in folio 2v or in the previous or following folio). The map in the centre of the figure is the enlarged corresponding section of the georeferenced map. Each point corresponds to a surviving head of household (green points) or to a registered death (larger red points), and the points outside *Fermerot* Street have been erased. The "gone" (4 in folio 1v and 1 in folio 2v) do not appear in the map, as they were not taken into account in this work. The putative path of the tax collector starts from *Fermerot* gate (below the North indication), goes towards south along the western side of the street, lined by the *Clairvaux* abbey *enclosure*, turns at the end of the street, and returns along the eastern side of *Fermerot* Street towards the north. The rectangles indicate the households included in the corresponding folio of the *marcs* tax register. Folio 2v and the corresponding eastern side are inside the cluster of higher grouped death relative risk, whereas folio 1v and the western side are outside (Fig 2). The western side of *Fermerot* Street at least partially corresponded to an area where the *Clairvaux* abbey rented houses in its *enclosure*, where housing density might have been lower.

Thus, taking into account deaths that were in all probability closely associated in space allowed us to evidence an uneven spatial distribution of the numerous deaths of 1400. In the same street, both sides could be affected by markedly different mortality patterns, as is the case for *Fermerot* Street (Fig 3).

#### Death and tax level

Higher mortality clusters stood predominantly in the wealthy northern part of the city whereas lower mortality clusters were located in the southern part where most low-tax taxpayers lived (compare Fig 2 with Fig 1). As a matter of fact, the mean tax level of the heads of households located within the formers was superior to that of their fellow citizens located within the latters (respectively 11.8 and 6.9 sols/taxpayer; Student's t test, p < 0.02).

We also examined mortality within the tax level-based clusters. The large cluster where high-tax payers were in excess (cluster 1 of Fig 1) included the larger area of higher mortality. In this cluster, the death rate was not higher than in the rest of the city (16.5% as compared to 15.9%), possibly because only 16% of its households stood within the higher mortality area. The cluster where low-tax payers were in excess (cluster 4 of Fig 1) included most of the areas of lower mortality, where stood 49.6% of its households. In this cluster, the death rate was lower than in the rest of the city (13.1% as compared to 17% outside the cluster; chi-square test, p = 0.04). Although of borderline statistical significance, this is in keeping with the previously mentioned data and suggests a spatial relationship between a higher proportion of low-tax payers and a lower death rate.

We thus tested whether low-tax payers died differently from the other heads of households. To this aim, we took into account the register for the previous year. The 325 heads of household deceased in 1400 were recorded in the register for 1399 (the other 5 dead persons of 1400 were not heads of households). The same register mentioned 1,548 out of the 1,715 survivors of 1400 (the other 167 survivors were newly registered for 1400). In 1399, among this total of 1,873 heads of households, 893 were low-tax payers. On the following year, their death rate was 21% as compared to 14% for the 980 heads of households who were not low-tax payers (Table 4). This pattern was also apparent within the mortality-based clusters. In the higher mortality clusters, low-tax payers died even more than their neighbours whereas in the lower mortality clusters, they nevertheless died in excess (Table 4). This suggests that the higher susceptibility of low-tax payers existed regardless of their home location.

**Table 4:** Deaths (in 1400) of low-tax payers and non-low-tax payers of 1399.

Mortality-based clusters	Higher	Higher	Lower	Lower	Dijon	Dijon
Tax level (1399)	Low	Non-low	Low	Non-low	Low	Non-low
Total (1400)	58	71	155	145	893	980
Deaths (1400)	28	20	30	10	188	137
p (chi-square)	< 0.05		< 0.002		< 10^−4^	
Death rate (%)	47.4%	28.2%	19.4%	6.9%	21%	14%

Legend: Line 1: Mortality-based clusters (or Dijon). Line 2: Tax level group (Low: low-tax payers in 1399; Non-low: heads of households who were not low-tax payers in 1399). Line 3: total number of households (deaths and survivors) in 1400. Line 4: number of deaths in 1400. Line 5: result of the chi-square comparing the low-tax payers and the non-low-taxpayers. Line 6: death rates as percent. Columns 2 & 3: pooled data from the 3 clusters of higher grouped death relative risk in 1400. Columns 4 & 5: pooled data from the 2 clusters of lower grouped death relative risk in 1400. Columns 6 & 7: data for the whole city.

A topographic factor influencing survival was thus superimposed on the susceptibility linked to the socio-economic status. One such a factor may be linked to the low housing density in the southern part. Indeed, the area of lower mortality centered by *Potet* Street was contiguous to a large estate of *Saint-Etienne* abbey that was not yet built, while that centered by the *Crais* still included vineyards **S9 Text**.

#### Death and profession

We tested whether heads of households exerting specific occupations were more (or less) affected during the epidemic. This was the case of one single professional group, bakers and millers. Their death rate (18.5%) was higher than that of the other heads of households whose profession was known (15.2%) (chi-square test, p < 0.02) while the death rates of the other professional groups were unremarkable **S1 Table.**

We also examined whether the uneven space distribution of certain professions altered death rate in the corresponding areas. This was the case of one single professional group, butchers. Twenty of the 22 butchers listed in 1400 lived in a restricted area defined by a cluster of 73 households (RR = 267; p < 10^−9^). This area stood between the Suzon River and the downgraded fortification of the *castrum* (Fig 2). It corresponded to the *Bourg* district where historical sources locate livestock slaughter and the meat trade, both performed under insanitary conditions that were condemned by contemporaries. In this area, one in four heads of households died (19 out of the 73 members of the cluster, as compared to 311 among the 1,972 settled outside the cluster; chi-square test, p < 0.02). In contrast, mortality was not increased in three other areas sharing spatial and/or professional characteristics with the *Bourg* district (results not shown). One of them was the contiguous area around *Forges* Street (Fig 2) where most knife, sword and spur makers were settled. Another one was an overlapping larger area where meat bakers were more numerous. A third one was the extramural area along the *Ouche* River where tanners and fishmongers were grouped, in a comparable riverside location **S9 Text**. This strongly suggests that the higher death rate of the *Bourg* district was linked to the activity of butchers.

### Higher mortality in the economically active centre in 1428

#### The 1428 epidemic

In 1428 a new epidemic struck the city, and the crude mortality rate rose from 30 per thousand in 1427 to 80 per thousand in 1428 (Table 1). At this time, the political and fiscal context was very different from that at the beginning of the century. The *Armagnacs* were at war with the duke of Burgundy and devastated the vicinity of Dijon **S3 Text.** This situation had economic and human consequences such as the recurrent levying of "fortification" taxes, limitations on trade, and many families having to host their fleeing relatives. As the number of households progressively declined (to reach a nadir of 1,284 in 1423), the authorities instituted a generalized increase in the *marcs* tax and added new fiscal households (the appointment of a new tax collector was followed by two dramatic rises in the number of newly registered households). In the same line, deaths of numerous household members other than the head were recorded in the register for the 1428 epidemic, implying a more exhaustive search for substitute taxpayers for the deceased. Only in this year were the concomitant deaths of children mentioned **S4 Text.**

This pattern led to a high number of multiple deaths and to a total number of recorded deaths that nearly equaled the year 1400. In addition, the register indicated numerous examples of family disruption, such as the surviving spouse "begs his/her bread" or "serves one's master", and an unusual number of departures from the city.

In 1428 as in 1400, the low-tax payers (individualized on the basis of their previous year tax level) died in excess. The register for 1427 mentioned 142 of the dead heads of households and 1,536 of the survivors of 1428. Among those 1,678 heads of households, 684 were low-tax payers in 1427: on the following year, 72 of them died, corresponding to a death rate of 11%, as compared to 7% for the other taxpayers of 1427 (chi-square test, p < 0.01).

#### Cartography of death

**Fig 4:**
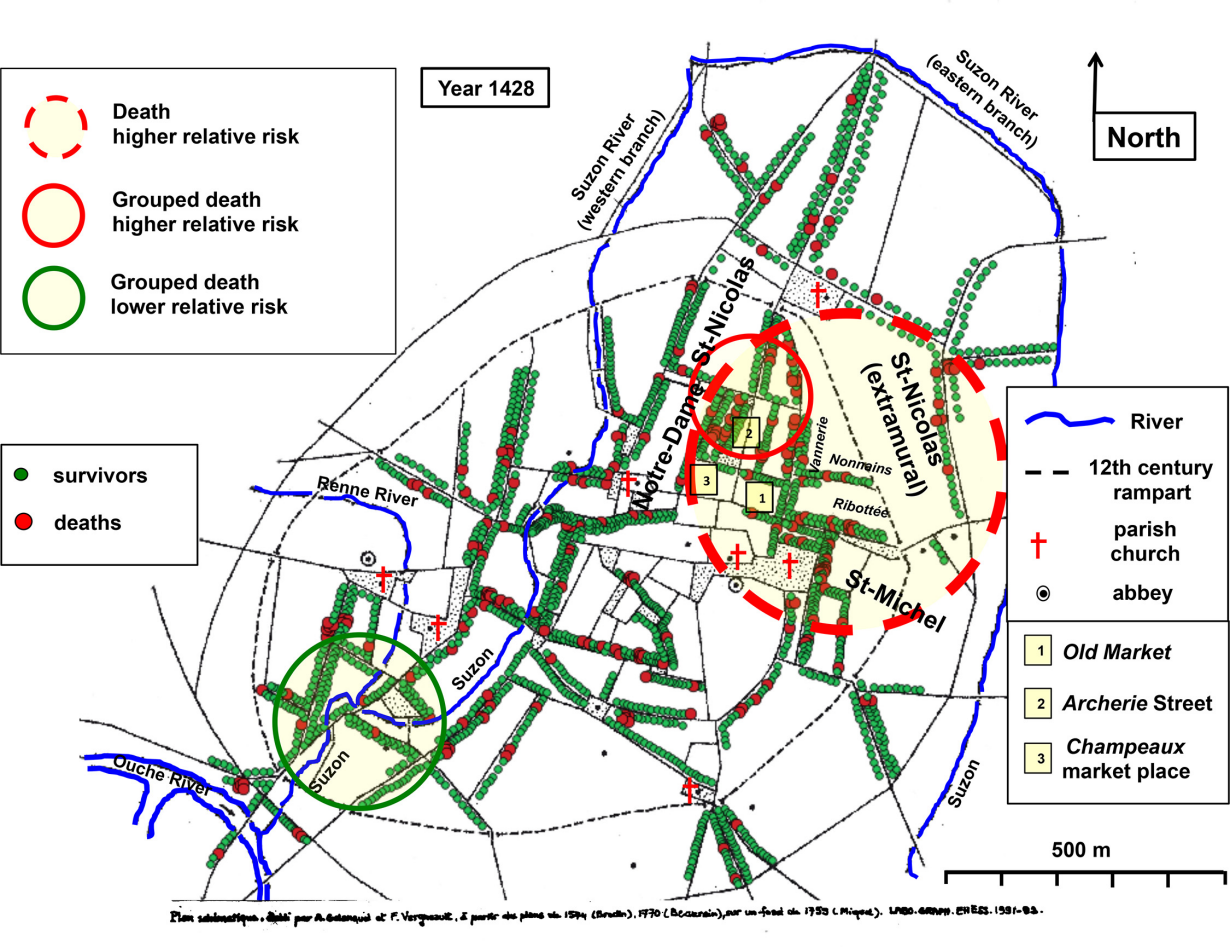
Cartography of death in 1428. Georeferenced map of medieval Dijon. Rivers as blue lines. Parish churches, abbeys and a number of prominent places are indicated. The 12th century rampart is shown by dotted lines. Each point corresponds to a surviving head of household (green points) or to a registered death (larger red points). Yellow area circled in dotted red line: spatial cluster where the relative risk of death doubled. Yellow area circled in solid red line: cluster with a higher relative risk of grouped death. Yellow area circled in green: cluster with a null relative risk of grouped death. Historical evidence of the location of the statistically significant clusters is presented in **S10 Text**.

In 1428, spatial analysis disclosed a large cluster of higher relative risk of death (Fig 4) associating 405 households with 81 registered deaths corresponding to a doubled relative risk (RR = 1.97, p = 0.0067). Most of the population present in the spatial cluster lived within the walls, in the economically active and wealthy space around the *Old Market*. There wheat was sold, the crop market was located, and bakers, drapers and second-hand clothing dealers proposed their products **S10 Text**. In the north of this area, stood a smaller cluster of higher relative risk of grouped deaths, associating 117 households with 28 grouped deaths (RR = 3.87; p < 0.001). This small cluster was mostly included within the large one and its location was nearly identical to that of the northern 1400 cluster of higher mortality (compare Fig 4 with Fig 2). In this more limited area, one out of four heads of households died. Among the most affected within the area were the rich inhabitants of *Archerie* Street at the southern end of which the new *Champeaux* market place was settled two years earlier **S10 Text**. In contrast, in the southwest, grouped deaths were absent in a cluster of 180 households (RR = 0; p = 0.0014).

Statistical analysis of the distribution of deaths within and outside the clusters confirmed that the intramural northern part of the city was affected by a higher mortality (Table 5). Within the cluster of higher relative risk of deaths, the death rate was, as expected, higher than in the rest of the city. Within the cluster of higher grouped death relative risk, the proportion of deaths (grouped and non-grouped) was significantly higher than in the rest of the city. Most of the inhabitants of the intramural parts of *Saint-Nicolas* and *Saint-Michel* parishes (70%) were within the large cluster of higher relative risk of death (Fig 4). Their death rate was significantly higher than that of the other parishes. In contrast, the proportion of deaths (grouped and non-grouped) was significantly lower within the southwestern cluster of lower grouped death relative risk.

**Table 5:** Excess deaths in the northeast of intramural Dijon in 1428.

Spatial entity	Higher death	Higher grouped death	Intramural *Saint-Nicolas* & *Saint-Michel*	Lower grouped death	Dijon
Total	405	117	444	180	1,930
Deaths	81	31	71	7	236
p (chi-square)	< 10^−7^	< 10^−5^	< 10^−2^	< 10^−3^	X
Death rate	20%	26.5%	16%	3.9%	12.2%

Legend: Line 1: Spatial entity. Line 2: total number of households (deaths and survivors). Line 3: number of deaths (grouped and non-grouped). Line 4: result of the chi-square comparing the group with the rest of the city. Line 5: death rate as percent. Column 2: data from the higher death relative risk cluster. Column 3: data from the higher grouped death relative risk cluster (mostly included in the previous one). Column 4: pooled data from the intramural parts of the northeastern *Saint-Nicolas* and *Saint-Michel* parishes (that largely corresponded to the higher death risk cluster of column 2). Column 5: data from the southwestern lower grouped death relative risk cluster. Column 6: data for the whole city. For each group, data of the control (rest of the city) can be computed by subtraction from the data of the whole city.

#### Death and profession

In 1428, a relationship between death and occupation was statistically significant for one single profession, winegrowers. The death rate of winegrowers was half that of the other heads of households whose profession was known (chi-square test, p < 0.01). This difference was already apparent in 1400 (but not statistically significant because of the small number of heads of households identified as winegrowers at the beginning of the century) and was also statistically significant in 1439 **S1 Table**. Spatial analysis showed that winegrowers preferentially resided in the south or outside the walls, and were scarce in the intramural part (results not shown). The few winegrowers who lived in the area of higher mortality had a death rate comparable to that of their neighbours and higher than that of winegrowers living in the rest of the city (respective death rates of 8.3% and 1.7%; Fischer exact test, p = 0.03). This strongly suggests that the relative resistance of winegrowers was the consequence of their housing geography rather than the professional activity itself.

### Contrasting topography of the mid-century epidemic (1438–1440)

#### The 1438–1440 epidemic

The epidemic that started in 1438 generated an excess of death in three successive registers, with respective crude mortality rates of 61 per thousand, 117 per thousand and 45 per thousand and a cumulative number of deaths roughly equivalent to that of the beginning of the century (Table 1). This probably reflected a more prolonged epidemic and information from inventories of inheritance suggest that deaths peaked in the late summer of the first two years. The epidemic affected a population weakened by several years of major starvation at a time when the *Ecorcheurs*, armed gangs wandering as a consequence of the Anglo-French war reached the city walls of Dijon **S3 Text**. The number of households that had progressively decreased in the preceding years dropped still further during the first two years of the epidemic. However, a rapid and lasting recovery began in the last year of the epidemic, with large numbers of newly registered heads of households, in conjunction with the return of security and the general economic improvement of the mid-15th century. Conversely to the two previous epidemics, the low-tax payers, possibly already decimated by starvation, had no demonstrable increased death rate (among the 796 low-tax payers of 1437, the death rate in 1438 was 7.7%, as compared to 5.7% for the 795 heads of households who were not low-tax payers in 1437, a non statistically significant difference).

#### The first year of epidemic

**Fig 5:**
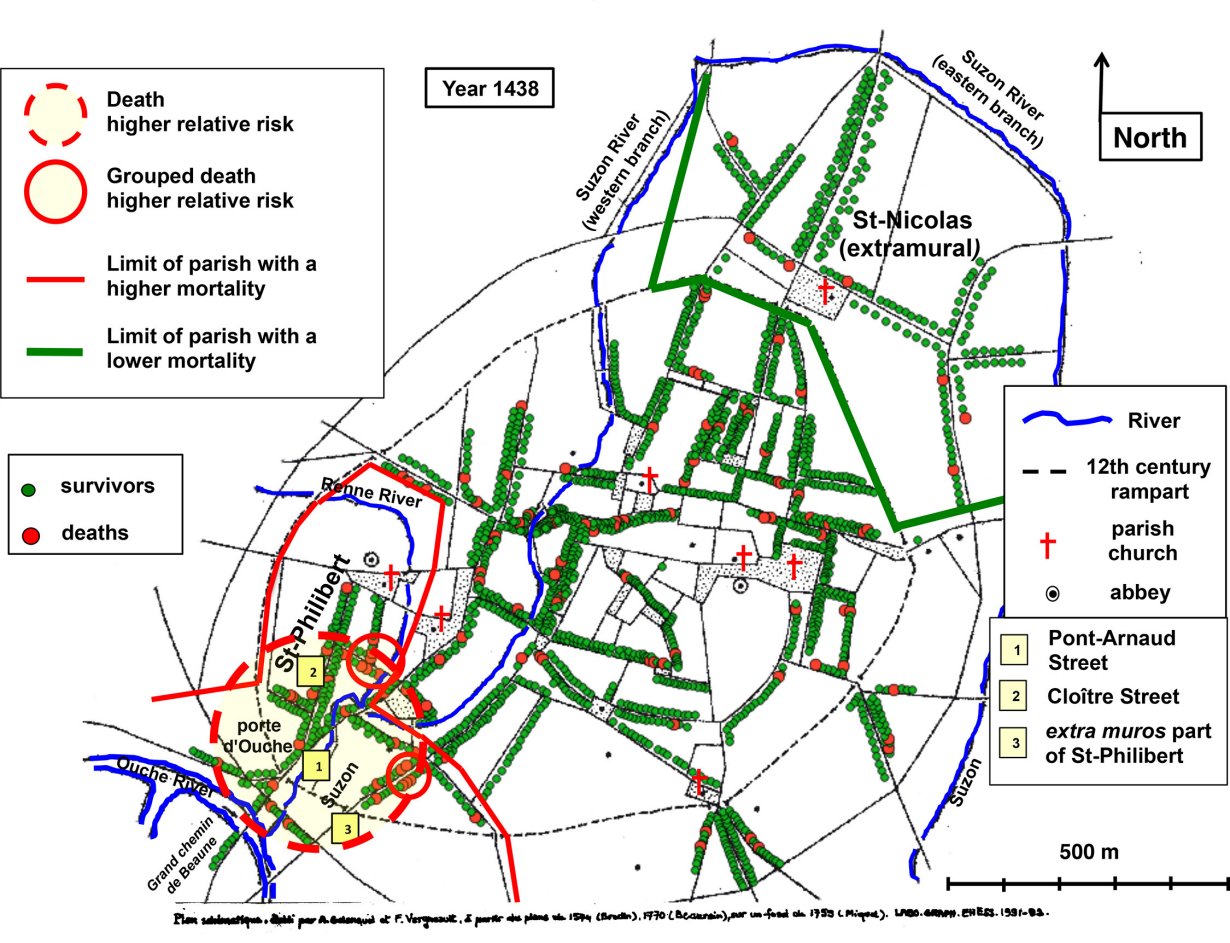
Cartography of death in 1438. Georeferenced map of medieval Dijon. Rivers as blue lines. Parish churches, abbeys and a number of prominent places are indicated. The 12th century rampart is shown by dotted lines. Each point corresponds to a surviving head of household (green points) or to a registered death (larger red points). Yellow area circled in dotted red line: spatial cluster with a higher relative risk of death. Yellow areas circled in solid red line: clusters with a higher relative risk of grouped deaths. The limits of the parish with a higher mortality (*Saint-Philibert*) are in red. The limits of the parish with a lower mortality (extramural part of *Saint-Nicolas*) are in green. Historical evidence of the location of the statistically significant clusters is presented in **S11 Text**.

In 1438, during the first year of this new epidemic, spatial analysis revealed a cluster of higher relative risk of death (Fig 5) involving 177 households with 28 reported deaths, corresponding to a threefold increase in relative risk (RR = 2.95, p = 0.028). In the north and east of this area, (and mostly included within it) stood two small clusters of higher grouped death relative risk (RR = 31.46 & RR = 34.98; p < 0.0001 & p < 0.001). This more affected area, in the southwest of the city, was entirely situated in *Saint-Philibert* parish, on either side of the *Ouche* gate, the southern entry into Dijon. Centred by the western branch of the *Suzon* River, it extended from the confluence with the *Renne* River to the confluence with the *Ouche* River. Ten years before, this area was characterized by a lower mortality (compare Fig 5 with Fig 4).

Statistical analysis confirmed that the southwestern part of the city was affected by a higher mortality (Table 6). Within the cluster of higher death relative risk, the death rate was, as expected, higher than in the rest of the city. Within the small clusters of higher grouped death relative risk, death (grouped and non-grouped) affected more than one out of two households. Most of the inhabitants of the *Saint-Philibert* parish (74%) were within the large higher death relative risk cluster (Fig 5). Their death rate was double than that of the other parishes. The extramural part of *Saint-Nicolas* parish stood at the opposite northern end of the city. There, the death rate was half that of the other parishes, which suggests that the spread of the epidemic to this part of the city was delayed.

**Table 6:** Excess deaths in the southwest of Dijon in 1438.

Spatial entity	Higher death	Higher grouped death	*Saint-Philibert* parish	Extramural *Saint-Nicolas*	Dijon
Total	177	19	237	216	1,799
Deaths	28	11	30	7	115
p (chi-square)	< 10^−7^	< 10^−14^	< 10^−3^	< 0.05	X
Death rate	15.8%	57.9%	12.7%	3.2%	6.4%

Legend: Line 1: Spatial entity. Line 2: total number of households (deaths and survivors). Line 3: number of deaths (grouped and non-grouped). Line 4: result of the chi-square comparing the group with the rest of the city. Line 5: death rate as percent. Column 2: data from the higher death relative risk cluster. Column 3: pooled data from the 2 higher grouped death relative risk clusters (included in the previous one). Column 4: data for the southwestern *Saint-Philibert* parish. Column 5: data for the extramural part of the northeastern *Saint-Nicolas* parish. Column 6: data for the whole city. For each area data of the control (rest of the city) can be computed by subtraction from the data of the whole city.

#### The second year of epidemic

In 1439, during the second year of the epidemic, when the deaths were at their maximum, spatial analysis did not indicate that they were unevenly distributed.

#### The third year of epidemic

**Fig 6:**
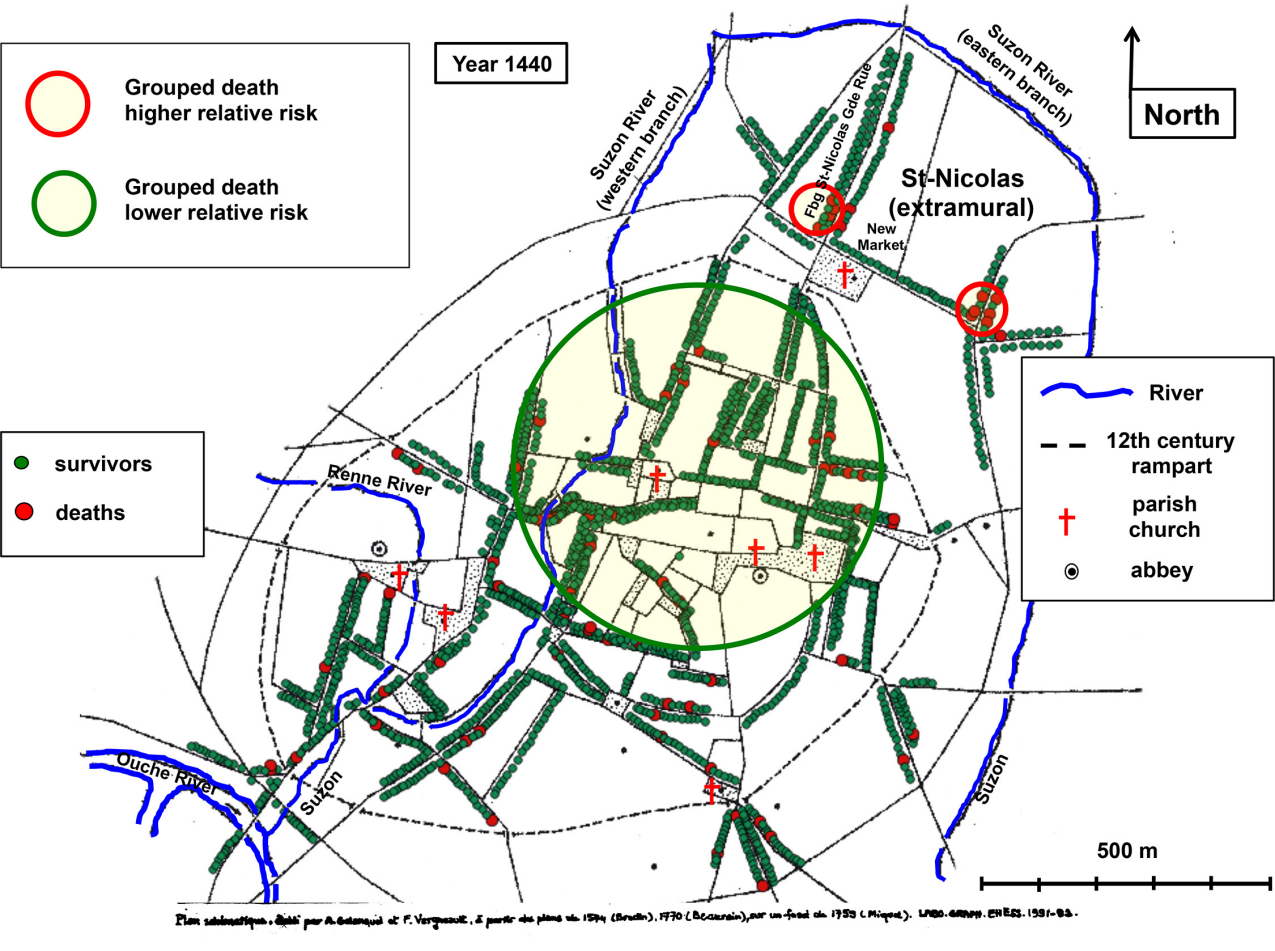
Cartography of death in 1440. Georeferenced map of medieval Dijon. Rivers as blue lines. Parish churches, abbeys and a number of prominent places are indicated. The 12th century rampart is shown by dotted lines. Each point corresponds to a surviving head of household (green points) or to a registered death (larger red points). Yellow areas circled in solid red line: clusters with a higher relative risk of grouped death. Yellow area circled in green: cluster with a null relative risk of grouped death. Historical evidence of the location of the statistically significant clusters is presented in **S11 Text**.

In 1440, during the third year (Fig 6), two small clusters of higher grouped death relative risk were found in the northeast of the city, in the extramural part of *Saint-Nicolas* parish (RR = 92.77 & RR = 85.03; p <10^−5^ & p = 0.014). In contrast, no grouped death was reported in a large cluster (involving 750 households) located in the northern and central part of the city (RR = 0; p = 0.021).

Statistical analysis of the distribution of deaths within and outside the clusters confirmed that clusters correspond to groups of households with contrasting mortality patterns (Table 7). In the two clusters of higher grouped death relative risk, the proportion of deaths (grouped and non-grouped) was significantly higher than in the rest of the city (Fischer exact test, p < 10^−9^). The reverse was true for the large cluster of lower grouped death relative risk (chi-square test, p < 10^−2^).

**Table 7:** Deaths in the mortality-based clusters in 1440.

Grouped death clusters	Higher RR	Lower RR	Dijon
Total	13	750	1,818
Deaths	9	25	78
p	Fischer test, p < 10^−9^	Chi-square test, p < 10^−2^	X
Death rate	ND	3.3%	4.3%

Legend: Line 1: Grouped death clusters (or Dijon). Line 2: total number of households (deaths and survivors). Line 3: number of deaths (grouped and non-grouped). Line 4: result of the statistical test comparing the cluster(s) with the rest of the city. Line 5: death rate as percent. Column 2: pooled data from the 2 clusters of higher grouped death relative risk. Column 3: data from the cluster of lower grouped death relative risk. Column 4: data for the whole city. ND: not done. For each cluster, data of the control (rest of the city) can be computed by subtraction from the data of the whole city.

## Discussion

We showed that the spatial analysis of deaths reported during "years of plague" in a medieval city can identify areas of higher or lower mortality and we discussed the factors that might underlie these uneven distributions in space. We based our study on tax registers that list and locate the heads of households. In contrast to exceptional documents such as the unique Givry parish register [23] tax registers generally omit other members of the families and those more miserable inhabitants who were not liable to taxation. Another limitation of our document source is a lack of information about the putative cause of death that is, in contrast, indicated in the Florentine *Gracia morti* books of the same period [6]. We assumed that in a given "year of plague", the spatial distribution of reported deaths represented that of the epidemic. The validity of this assumption is supported by the similar conclusions drawn when the analysis is restricted to deaths grouped in space, either in the same household or in contiguous or nearly contiguous households. The presence of grouped cases within and in the vicinity of the homes of diseased persons, suggestive of epidemic mortality, is demonstrated in medieval as well as modern outbreaks of plague [6, 24, 25].

In the absence of more precise historical indication of the symptoms of the disease(s) or of molecular characterization of the causative agent(s), the nature of the infection(s) cannot be formally determined. In early 15th century Dijon, the single historical source alluding to "contagious bubos", a strong basis for the characterization of plague from historical sources [26] refers to the epidemic of 1414 [27], a year for which no *marcs* tax register is available. In view of this limitation, our results cannot contribute to the comparison between medieval and modern outbreaks of plague [7, 25, 28, 29]. Nevertheless, aside from their historical interest, the results may have implications for a better understanding of the spread of epidemics in the absence of medical treatment.

The preservation of the urban topography and the availability of numerous historical sources allowed for the establishment of a reliable GIS-based map of medieval Dijon, particularly for the intramural parts where the greatest differences in mortality occurred. The individual georeferencing of heads of households is more arbitrary. Each household was positioned along streets corresponding to their modern equivalents, following the reconstituted path of the tax collector, in the order of their recording in the registers. Although the repetitious sequence of households recording by the clerks to collect this secular tax may represent another example of unwritten cartography [30], this choice is based on an oversimplification of the implementation of the annual census in medieval streets that were not linear. Nevertheless, transforming the sequence of households listed in the registers into two-dimensional data on a map illustrates the proximity of individuals living in neighbouring streets or the distance between individuals living at the extremities of the same street. These spatial relationships between individuals probably play a role in the spread of epidemic diseases, either by human-to-human transmission or through the impact of the shared urban and/or socio-professional characteristics of the area.

Although our study was based on tax registers unique in their continuity over time and the accuracy of their topographic information and took advantage of the preserved geography of the historical center of Dijon, our approach presents a number of limitations. The population studied did not completely correspond with the victims of the epidemic as not every death was due to the communicable disease and we had no information about a large part of the population at risk. The positioning of households on the georefrenced map was in part arbitrary, and corresponded to the search of spatial relationships between individuals rather than to a reconstitution of the urban space in itself. However, when mortality-based clusters approximately corresponded with parish(es), in 1428 and 1438, these latter displayed comparable mortality patterns, showing that, at least in these cases, the cartography generated by the individual georeferencing of heads of households matched with a traditional partition of the urban space. With these limitations in mind, our analysis was based on the following assumptions: (i) the position of households in the registers is indicative of a spatial relationship between the corresponding homes; (ii) the spatial distribution of reported deaths is representative of that of epidemic mortality; (iii) deaths grouped in space are more likely due to an epidemic.

For each of the three epidemics, spatial analysis reveals singularities that may reflect the characteristics of the infectious disease, as modified by historical context, fiscal practice and climatic conditions [31].

The epidemic that struck Dijon in 1400 corresponds to a "brief, violent and generalized" European mortality crisis [32] that in all probability represents a major recurrence of the plague [10]. The time of death, indicated for the second year of epidemic, is compatible with the late summer or autumnal seasonality of medieval plagues [6, 33, 34]. Millers and bakers were more affected and the meat trade district where livestock were slaughtered exhibited an increased mortality, in keeping with the demonstrated relationships between the plague and professional activities [24]. This fits with the higher densities of rat remains in the vicinity of crop stocks, bakeries and slaughterhouses reported from archaeological studies of plague sites [35]. The direct impact of these professional activities on mortality was not apparent during the other two epidemics, possibly because their mortality rates were lower or because they were caused by different diseases. Alternatively, this difference may reflect an improvement in urban conditions, such as the street paving that was in progress, or the effect of putative sanitary decrees. Historical sources mention sanitary orders concerning barbers (discarding blood from bloodletting within the city was prohibited in 1401) but those concerning butchers (the transient relocation of meat stalls from the *Bourg* district to crossroads and city squares in view of "the danger of plague in the *Grand Bourg*") are found only in the next century.

In contrast to the 1400–1401 epidemic that raged throughout Europe [10, 32], the other two epidemics were more localized and may represent "minor plagues" or other diseases. Nevertheless, the 1428 mortality pattern shared several characteristics with that of 1400. Although the death toll of the second epidemic was lower, the number of recorded deaths was of the same magnitude, thanks to a more exhaustive recording. In both cases, the low-tax payers were more vulnerable to the epidemics, in agreement with the higher susceptibility of the poor shown in most studies [6, 24]. In 1400, as well as in 1428, there were clusters of higher mortality in the wealthiest northern part of the city, where most commercial and craft activities were concentrated, whereas lower mortality clusters occurred in the southern part. In a city where garbage collection was left to the inhabitants, the proximity of food stocks, crop market, second-hand clothing dealers (in the *Old Market* area and the *Champeaux* market place) and/or an open area used for slaughter (the *Bourg* district), created conditions suitable for rats to pullulate and favored disease transmission by fleas [35] or by lice [36]. Again, these characteristics are compatible with those of a plague [11, 24]. They differ from what was reported during the 1400 epidemic in Florence, where the wealthier neighbourhood at the centre (where the central grain market was located) had a lower mortality rate than the poor parishes on the periphery [28]. This discrepancy may reflect the exceptional sanitary conditions in Florence [6], as exemplified by the completion of street paving in 1399 [37], whereas the paving of Dijon required a special tax in 1428 [38]. Another characteristic of the northern part of Dijon is a higher housing density (still apparent on the 1812 cadastral map) that provides ecological niches for black rats and favors disease transmission by ectoparasites. In contrast, areas of low housing density in the south were relatively spared. Winegrowers, most of whom lived outside the area of concentrated housing and worked at a walking distance in the countryside, where a rat's aversion for open areas [35] would apply, were relatively spared. These characteristics suggest that in 1428 as well as 1400, the inhabitants of Dijon were victims of recurrences of plague.

The epidemic that started in 1438 differed from the previous ones in several respects. An excess mortality was reported over three years, with late summer seasonal peaks suggesting that it was not caused by the influenza that raged "throughout France" in the same year [32]. A minor climatic change initiated a cooler period from the mid-1430 years [31] and several years of starvation might have altered the resistance to infectious disease(s). Contrary to the previous epidemics and with what is reported elsewhere, the low-tax payers had no demonstrable increased death rate. However, the registers may not include the more miserable, those already dead or excluded from taxation after several years of starvation. In Dijon, another peculiar feature of the 1438–1440 years is the threat from the armed gangs of *Ecorcheurs*, who "spread… diseases that originate from poverty and corpse pestilence" [39]. Although the precise mapping of epidemic spread, as first used for the London 1854 cholera epidemic and applied to the deaths from plague in 1430 Florence [6] is impossible in this case, sequential spatial analysis suggests variation in the geography of mortality. When mortality exhibited a spatial heterogeneity, as in the first and the third years, the areas of higher relative risk of death were, respectively in the southwest and in the northeast. These locations may suggest a waterborne disease or an infection brought from the south for the southwest area and the occurrence of residual cases of a waning epidemic in a northeastern extramural area, when the inner part of the city was already disease-free. Taken together, these data suggest that the 1438–1440 epidemic was caused by a disease different from the two previous ones.

## Conclusion

Using GIS-based quantitative analysis, we confirm the socio-economic dichotomy in medieval Dijon, between a wealthier and more economically active northern part and a southern part where low-tax payers were more numerous [20, 21]. We show that, during the major recurrence of the Black Death in 1400, areas of higher mortality were present in the former whereas areas of lower mortality were in the latter. The low-tax payers, although more at risk of death from the epidemics, were relatively protected when they lived in the south, suggesting that urban characteristics rather than the individual socio-economic level of the inhabitants determined the geography of mortality. A high concentration of housing and the proximity to food stocks in the northern intramural part of the city may have created suitable conditions for rats to pullulate. A comparable topography is demonstrated for the minor, more local, epidemic of 1428. The epidemic that started a decade later lasted for 3 years and had a different and possibly evolving geography. During the first year, cases were concentrated around the southern gate, at the confluence of three rivers while deaths during the second year were evenly distributed. In the last year, residual foci of deaths persisted in the northern suburb. Our results fit with the view that the generalized 1400–1401 epidemic was a plague and suggest that this was also the case in 1428. The geographical pattern of the 1438–1440 epidemic may suggest that a different, possibly waterborne, disease was involved.

In the recent years, progress in various scientific fields has improved our knowledge of historical epidemics [40], by characterizing the infectious agents [13, 14, 15, 16, 17], the influence of climate [41], the geographic origin [42] and health condition [43] of the deceased and of the surviving population [44]. The availability of GIS has renewed our approach to epidemiological surveillance and research. As far as we know, its application to historical research on mortality and diseases has been limited and has focused on modern history [45]. The data presented here outline the potential for and the interest of GIS-based spatial analysis of medieval epidemics, providing that appropriate source materials are available.

## Supporting Information

S1 Fig Heads of household annual mortality rate and "years of plague". (PDF)

S2 Fig Deaths of 1400 in the *marcs* tax register. (PDF)

S1 Table Members of selected professions. (DOCX)

S1 Text Historical sources and database. (DOCX)

S2 Text Registered heads of households and actual population. (DOCX)

S3 Text Historical sources and "years of plague". (DOCX)

S4 Text Counting the deaths. (DOCX)

S5 Text Exempted heads of households. (DOCX)

S6 Text Low-tax payers. (DOCX)

S7 Text Cartography and georeferencing. (DOCX)

S8 Text Statistical analysis. (DOCX)

S9 Text Historical evidence and the 1400 clusters. (DOCX)

S10 Text Historical evidence and the 1428 clusters. (DOCX)

S11 Text Historical evidence and 1438 and 1440 clusters. (DOCX)
