# Supplementary figures and images for: Historical Epidemics Cartography Generated by Spatial Analysis: Mapping the Heterogeneity of Three Medieval "Plagues" in Dijon
Source: PLoS One. 2015 Dec 1;10(12):e0143866. doi: 10.1371/journal.pone.0143866 (PMC4666600; doi:10.1371/journal.pone.0143866)

## Heads of household mortality rate and "years of plague"

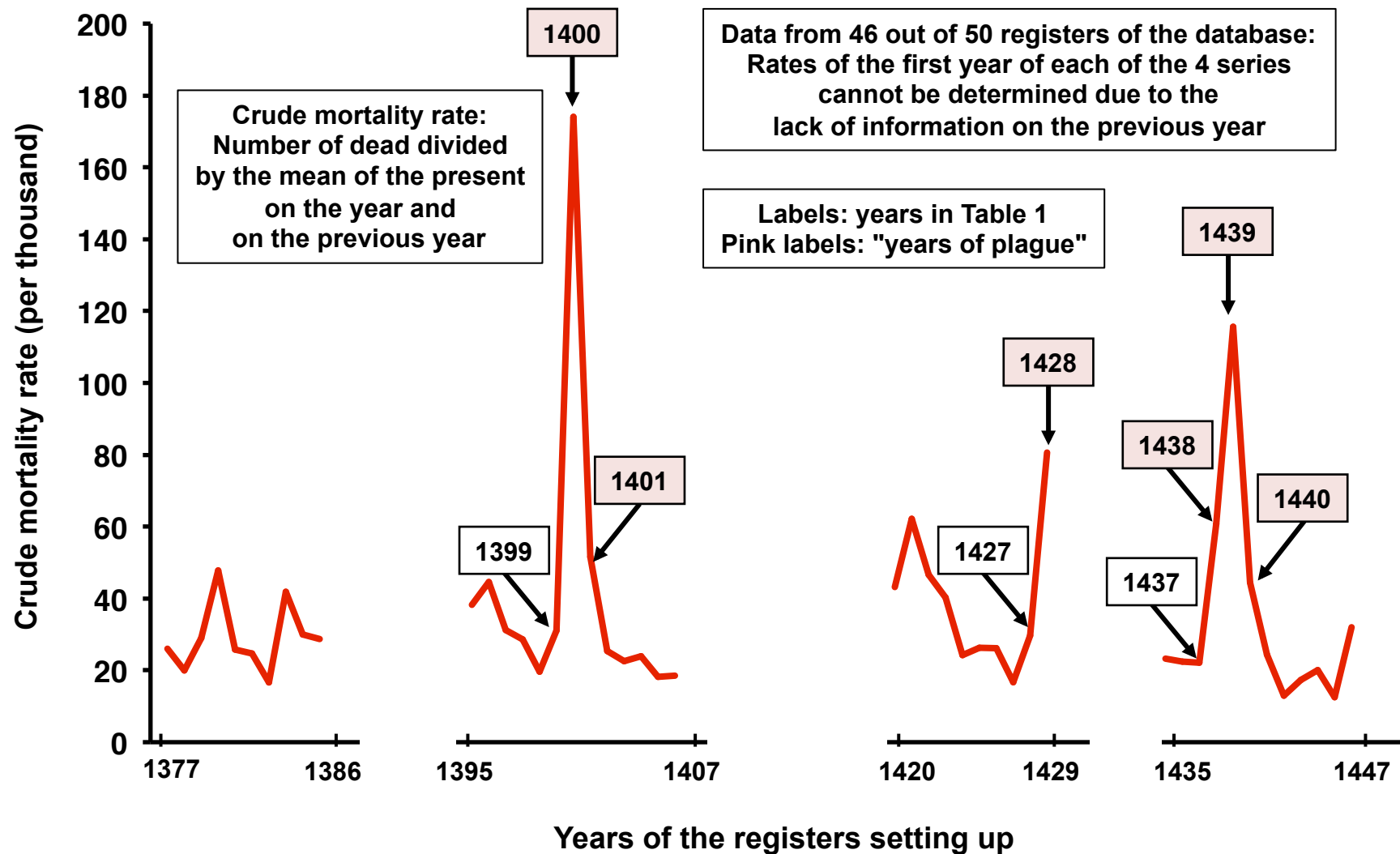

Supplement: S1 Fig — (PDF) [file pone.0143866.s001.pdf]
