# Supplementary material for: Historical Epidemics Cartography Generated by Spatial Analysis: Mapping the Heterogeneity of Three Medieval "Plagues" in Dijon
Source: PLoS One. 2015 Dec 1;10(12):e0143866. doi: 10.1371/journal.pone.0143866 (PMC4666600; doi:10.1371/journal.pone.0143866)

Deaths of 1400 in the *marcs* tax register

[ADCO, B11490, 1401, folio 2v]

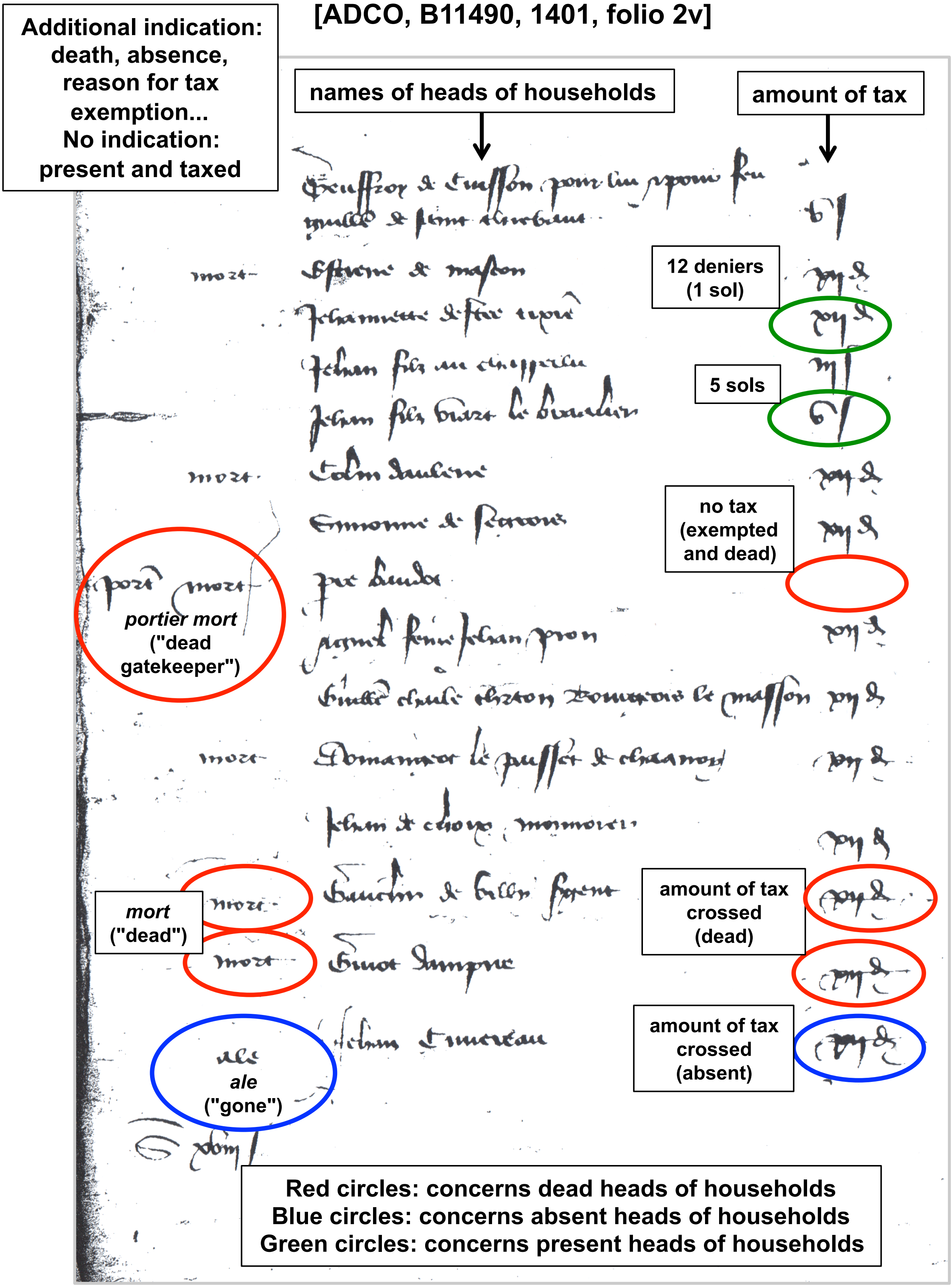

Supplement: S2 Fig — (PDF) [file pone.0143866.s002.pdf]
