# Supplementary material for: Historical Epidemics Cartography Generated by Spatial Analysis: Mapping the Heterogeneity of Three Medieval "Plagues" in Dijon
Source: PLoS One. 2015 Dec 1;10(12):e0143866. doi: 10.1371/journal.pone.0143866 (PMC4666600; doi:10.1371/journal.pone.0143866)
Supplement: S1 Table — (DOCX) [file pone.0143866.s003.docx]

**S1 Table. Members of selected professions**

|  | 1400  Total | 1400  Dead | 1428  Total | 1428  Dead | 1439  Total | 1439  Dead |
| --- | --- | --- | --- | --- | --- | --- |
| Known profession | 793 | 128* | 1264 | 85** | 1363 | 125** |
| Bakers & millers | 34 | 10* | 48 | 1 | 70 | 4 |
| Barbers & physicians | 12 | 1 | 28 | 4 | 36 | 7 |
| Butchers | 22 | 4 | 35 | 1 | 39 | 2 |
| Merchants, mercers & grocers | 41 | 4 | 50 | 4 | 45 | 6 |
| Metal craftsmen | 58 | 11 | 75 | 9 | 77 | 6 |
| Sewers | 75 | 9 | 67 | 8 | 67 | 5 |
| Shoemakers & cobblers | 46 | 7 | 70 | 10 | 52 | 4 |
| Tanners & fishmongers | 24 | 5 | 22 | 0 | 28 | 4 |
| Weavers, drapers & textile craftsmen | 61 | 12 | 94 | 2 | 111 | 19 |
| Winegrowers | 101 | 11 | 285 | 8** | 335 | 18** |

Total numbers and numbers of dead are indicated for the three major "years of plague". The number of heads of households whose profession is known from the sources is also indicated. Sewers and shoemakers & cobblers are indicated as examples of professionals who were numerous and settled diffusely in the urban space [19, p 348-50]. Tanners & fishmongers are grouped because they were settled in the same area. * Death rate significantly higher (chi-square test, p < 0.02). ** Death rate significantly lower (chi-square test, p < 0.01).
