# Supplementary material for: Historical Epidemics Cartography Generated by Spatial Analysis: Mapping the Heterogeneity of Three Medieval "Plagues" in Dijon
Source: PLoS One. 2015 Dec 1;10(12):e0143866. doi: 10.1371/journal.pone.0143866 (PMC4666600; doi:10.1371/journal.pone.0143866)
Supplement: S1 Text — (DOCX) [file pone.0143866.s004.docx]

**S1 Text. Historical sources and database**

The *marcs* tax was named from a silver currency unit that amounted 3 francs or 3 *livres* (pounds). The tax was established at the end of the 12th century as an annual global compensation for the granting of a city chart by the duke. From 1284 the global tax was replaced by an individualized contribution paid by the solvent heads of households members of the urban community. The total amount of the *marcs* tax was marginal in the duke's annual income [Rauzier J. [Budget and management of a principality in the 14th century: the Burgundy dukedom of Philip the Bald]. Comité pour l'histoire économique et financière de la France, editor. Paris: Imprimerie Nationale; 1996, p 245 & 699. French] and the information provided on the heads of households by the registers was probably of higher value for the power than the tax itself.

The *marcs* tax level of a given head of household was established and annually revised according to his/her assets within the city, resulting in annual registers. Heads of households not submitted to the *marcs* tax (either because they were not members of the urban community or for personal reason) were recorded in the registers without tax level and the reason for their lack of taxation was indicated.

Sixty-nine marcs tax registers, dated from 1357 to 1501, are preserved [*Archives Départementales de la Côte d'Or* (ADCO), B11483-B11501]. For the setting up of our database, we selected 50 annual registers grouped in 4 continuous series (separated by periods where the intermediate registers are not preserved) on the following years: 1376-1386 [ADCO, B11486-87], 1394-1407 [ADCO, B11488-91], 1419-1429 [ADCO, B11492-93] and 1434-1447 [ADCO, B11494-97].

The program, that takes into account the variability of medieval names, allowed the setting up of a single database for the 108,945 annual contributions of the 50 registers (a contribution corresponds to the annual registration of a head of household, regardless of his/her status (present, dead, absent...)). It allowed the individualization of 13,002 potential heads of households listed in one or several registers with a defined duration. When available, additional information obtained from other sources (registers for specific taxes, inventories of inheritance, tax relief requests...) were added [list and references in 21, p 52-53].
