# Supplementary material for: Historical Epidemics Cartography Generated by Spatial Analysis: Mapping the Heterogeneity of Three Medieval "Plagues" in Dijon
Source: PLoS One. 2015 Dec 1;10(12):e0143866. doi: 10.1371/journal.pone.0143866 (PMC4666600; doi:10.1371/journal.pone.0143866)
Supplement: S2 Text — (DOCX) [file pone.0143866.s005.docx]

**S2 Text. Registered heads of households and actual population**

The survivors taken into account in this work were the living individuals present when the register was established. The absent and those not corresponding to individuals (such as religious houses, "the vicar", "the heirs of"...) were excluded. During the 9 years studied, the total of these latter amounted 89 households (5 to 19 per year).

The number of heads of households indicated in the *marcs* tax registers is an underestimate of the inhabitants for the following reasons. (i) A given head of household was registered for his/her whole family (including in some cases servants). (ii) Although heads of households exempted from the *marcs* tax were recorded in the registers, the registration of these citizens not submitted to the tax was less exhaustive. (iii) The registers enroll essentially solvent heads of households, leaving aside most of the very poor, vagabonds and beggars, whose proportion, although difficult to figure out has been estimated to a few percent in medieval Paris [Geremek B. [The margins of society in late medieval Paris]. Flammarion: Paris; 1976, p 7. French, translated from Polish.]. The relationship between the number of households and the number of inhabitants varies according to places and demographic conditions [Blockmans W, Dubois H. [Times of crises (14th and 15th centuries)], in Bardet JP and Dupâquier J, editors [History of populations in Europe: 1 From the origins to the beginnings of demographic revolution]. Paris: Fayard; 1997, p 208. French]. A 3 to 5 factor is often proposed to evaluate the latter from the former. The estimate of a 10,000 population in the 14th to 15th century Dijon is based on such an assumption [Humbert F. [Municipal funds in Dijon from the mid-14th century to 1477]. Paris: Les Belles Lettres; 1961, p 24. French].
