# Supplementary material for: Historical Epidemics Cartography Generated by Spatial Analysis: Mapping the Heterogeneity of Three Medieval "Plagues" in Dijon
Source: PLoS One. 2015 Dec 1;10(12):e0143866. doi: 10.1371/journal.pone.0143866 (PMC4666600; doi:10.1371/journal.pone.0143866)
Supplement: S3 Text — (DOCX) [file pone.0143866.s006.docx]

**S3 Text. Historical sources and "years of plague"**

In 1400 and 1401, historical records [10] and numerous local source documents reflect the epidemics, such as the records from the city council indicating that the schoolmaster of Dijon "could not maintain his condition" because "numerous children died" [*Archives Municipales de Dijon* (AMD), B144, 1400-1401, 3v, quoted in [21] p 525].

Whereas the year 1428 does not correspond to a widespread recurrence of the Black Death, local sources are suggestive of an epidemic, as religious processions were hold "against the pestilence of epidemics" in July and September [AMD, E3 & B52, quoted in [19], p569]. In 1429, Perrin Quampenier submits a tax relief request justified by the fact that "he still owes 25 francs" for the farming of the *Poissonnerie* (fish market) on last year, which he cannot pay "in view of the mortality that... (unreadable) on that year" [AMD, B152, 1427-1431, quoted in [21], p 525].

In 1428, the epidemic occurred during years when the *Armagnacs,* supporters of the heir of the throne of France in the Anglo-French war, devastated Burgundy and the nearby Champagne [Vaughan R. Philip the Good: the apogee of the Burgundy. London: Longmans; 1970].

For year 1439, historical records [10] and local source documents allude to the epidemics, such as the mention that, in view of the "great mortality that was then in Dijon", the squire of the neighbouring village of Talant was prepared to house the Duke [ADCO, B6313, 83r].

The 1430s years were marked by a major starvation, attested by local sources ("one saw... the poor gathering on dunghills and die of hunger" [Dom Plancher. [General and particular history of Burgundy]. Dijon; 1739-1781, vol. 4, p 289. French]). Dijon was submitted to repeated attacks of the *Écorcheurs* ("skinners"). These armed gangs wandering as a consequence of the Anglo-French war, were "in front of the walls" in February 1438 and in the extramural part of *Saint-Nicolas* parish in March 1440 [Gras P. [History of Dijon]. Toulouse: Privat; 1981, p 77. French].
