# Supplementary material for: Historical Epidemics Cartography Generated by Spatial Analysis: Mapping the Heterogeneity of Three Medieval "Plagues" in Dijon
Source: PLoS One. 2015 Dec 1;10(12):e0143866. doi: 10.1371/journal.pone.0143866 (PMC4666600; doi:10.1371/journal.pone.0143866)
Supplement: S4 Text — (DOCX) [file pone.0143866.s007.docx]

**S4 Text. Counting the deaths**

Among the deaths of the 9 years analyzed, 90% were deaths of a head of household.

Reports of concomitant deaths in the same household are suggestive of epidemic mortality: among the 72 concomitant deaths in the same household reported in the 50 annual registers of the database, 48 occurred during the 6 "years of plague".

Counting the deaths from multiple deaths:

When a multiple death occurring in the same household involves the head of household and his wife it adds 2 deaths to the number of single deaths (this is the case for years 1400, 1401, and 1437-1440). For the 1428 epidemic, the register quotes 26 households with multiple deaths, corresponding to 11 deaths of husband and wife or of 2 sisters, 2 deaths of wife and children followed by the departure of the surviving husband and 13 deaths of husband, wife and children. Assuming a number of 3 children per nuclear family, this leads to 95 additional deaths (22 for double deaths, 8 for deaths of wife and children, 65 for 13 extinctions of nuclear families). Deaths of heads of households accounted for 33% of multiple deaths (as compared to 97% of single deaths).

Median number of children in nuclear families:

The estimate of 3 children in a nuclear family was based on the median number of children in 688 contemporary households within which the number of living children could be traced from several source documents [detailed in 21, p 423-424]. Lowering the median number of children to the underestimate of 2 per nuclear family does not modify the results of spatial analysis of the 1428 mortality in this work.
