# Supplementary material for: Historical Epidemics Cartography Generated by Spatial Analysis: Mapping the Heterogeneity of Three Medieval "Plagues" in Dijon
Source: PLoS One. 2015 Dec 1;10(12):e0143866. doi: 10.1371/journal.pone.0143866 (PMC4666600; doi:10.1371/journal.pone.0143866)
Supplement: S5 Text — (DOCX) [file pone.0143866.s008.docx]

**S5 Text. Exempted heads of households**

Exempted for poverty:

The number of exempted for poverty varied from 1 to 19 during the years studied. Although the instructions to the *marcs* tax collector stated that he had to "speak of the poor and put them as beggars" (ADCO, B11488, 1395, 94r), registered beggars did not entirely reflect the more underprivileged population. Rather than a taxation category that would be exempted on a stable basis, they represented selected heads of households whose insolvability was noticed by the clerk. With few exceptions, he will not make the effort to register them on the following year [21, p 383-384]. Although their homeless status was sometimes indicated, their ultimate fate was unknown.

Exempted by privilege:

The other exempted were grouped for analysis and qualified as exempted by privilege. They accounted for 4.2% to 5.7% of the households corresponding to individuals during the years studied. The number of registered nobles varied from 6 to 12. With the exception of a few ecclesiastics registered as individualized heads of households, most ecclesiastics were identified by their function, as priests of the 7 parishes, or as heads of religious houses (convents, houses in Dijon for abbeys of Burgundy, hospitals) and were excluded from our analysis. The number of heads of households exempted on an individual basis varied from 43 to 68 during the years studied. They benefited of a privilege (either as officers, servants or members of the entourage of the duke or as linked with one of the two major abbeys of Dijon) or exerted selected professions (physicians, notaries...) and were heterogeneous with regard to their wealth: some of them were outstanding officers or wealthy professionals while others were low-level servants of the duke.
