# Supplementary material for: Historical Epidemics Cartography Generated by Spatial Analysis: Mapping the Heterogeneity of Three Medieval "Plagues" in Dijon
Source: PLoS One. 2015 Dec 1;10(12):e0143866. doi: 10.1371/journal.pone.0143866 (PMC4666600; doi:10.1371/journal.pone.0143866)
Supplement: S6 Text — (DOCX) [file pone.0143866.s009.docx]

**S6 Text. Low-tax payers**

Although the very poor were not (or no more) recorded in the registers, the approximate half of tax payers charged at the 1 sol lower limit of the *marcs* tax can be considered as members of the poor population of Dijon. Their proportion corresponds to that of the taxpayers who were considered below poverty line in Lyon in the same period [Gonthier N. [Lyon and its poor in the middle ages (1350-1500)]. Lyon: L'Hermès; 1987, p 40. French] and of the day-to-day workers in the medieval town [Desportes P. [The city] in Favier J, editor [Medieval France] Paris: Fayard; 1983, p 201-215. French]. It should be pointed out that an annual 1 sol tax seems affordable for a winegrower (winegrower is the more frequently mentioned profession for low-tax payers): the daily salary of a winegrower amounted 1 to 2 sols at the end of the 14th century [Stella A. [The social profile of winegrowers in northern Burgundy from the 14th to the 18th century]. *Bulletin du Centre Pierre Léon d'histoire économique et sociale*. 1996; 3-4: 79. French] and 2 to 3 sols in the mid-15th century [Tournier C. [Notes about vineyard growing and winegrowers in Dijon between 1430 and 1560]. *Annales de Bourgogne*. 1952; 24: 151-152. French].
