# Supplementary material for: Historical Epidemics Cartography Generated by Spatial Analysis: Mapping the Heterogeneity of Three Medieval "Plagues" in Dijon
Source: PLoS One. 2015 Dec 1;10(12):e0143866. doi: 10.1371/journal.pone.0143866 (PMC4666600; doi:10.1371/journal.pone.0143866)
Supplement: S7 Text — (DOCX) [file pone.0143866.s010.docx]

**S7 Text. Cartography and georeferencing**

The map matrix was based on the earlier explicit maps of Dijon (the 1574 map of Bredin and the 18th century maps of Beaurain and Miquel) and on numerous published works [quoted in 21]. It took into account a number of preserved landmarks, the known situation of the surrounding walls, of the city gates and of major roads. The modern counterparts of medieval streets are precisely known for the intramural part of the city in view of the preservation of Dijon historical centre. They are more hypothetical for the suburbs that were destroyed when the city was besieged in 1513. The follow up of heads of households was taken into account in order to ascertain the minor variations of the streets names and/or boundaries during the period [Galanaud A, Galanaud P. Heads of households as tools for cartography of the late medieval Dijon. 10th International Conference on Urban History; 2010 Sept 2-4; Ghent, Belgium].

Locating heads of households:

The annual updating of the registers was based on an actual inquiry within the streets of Dijon [21, p 85]. With few exceptions, the registration of heads of households started from the *Fermerot* gate, on the western side of *Fermerot* Street, along the *Clairvaux* *enclosure* (the *Clairvaux* cellar still exists nowadays). In the absence of explicit landmark, the first head of household in a street was positioned in the continuity of the last one of the previous street. In a given street, the points were approximately equidistant along the line representing the street. When the points were numerous in a short street (as in several streets in the centre of the city) they were arranged in a quincunx under several layers. When the point density was low or when the limit of housing was not defined (as in most suburban streets) the points were spaced with a pattern comparable to that of neighbour streets, without trying to fill in the space. When the clerk registered successively two sides of a street, in the absence of explicit landmark, the first group of inhabitants was positioned on this street side that better respects the continuity with the previous street. For the third epidemic, that progressed over 3 years, the shapefile of the first year was used as a basis for successively realizing the other two annual shapefiles, in order to maintain the survivors in their (at least relative) positions. That the order of recording reflected a topographic pattern is supported by the following examples: when a head of household temporarily left the city or his home, most of his neighbours in the registers were the same when he came back; in the year following an epidemic, most new heads of household were settled in the gaps left by the deceased.
