# Supplementary material for: Historical Epidemics Cartography Generated by Spatial Analysis: Mapping the Heterogeneity of Three Medieval "Plagues" in Dijon
Source: PLoS One. 2015 Dec 1;10(12):e0143866. doi: 10.1371/journal.pone.0143866 (PMC4666600; doi:10.1371/journal.pone.0143866)
Supplement: S8 Text — (DOCX) [file pone.0143866.s011.docx]

**S8 Text. Statistical analysis**

The Kulldorff's method consists in applying a circular window over each household localized in the map and in incrementing its size to a maximum corresponding to 50% of the spatially analyzed population. The relative risk for each circle is calculated by comparing the prevalence of cases inside and outside the circle. The circle with the highest relative risk among all the circles is considered as the most likely. SaTScan then determines secondary clusters as non-secant clusters of successive lower relative risks. In order to test the null hypothesis (the differences in prevalence would reflect fortuitous sampling fluctuations) the deceased and survivors were 999 times commuted at random for every location, and each time the relative risk obtained "at random" was compared to the observed one.
