# Supplementary material for: Historical Epidemics Cartography Generated by Spatial Analysis: Mapping the Heterogeneity of Three Medieval "Plagues" in Dijon
Source: PLoS One. 2015 Dec 1;10(12):e0143866. doi: 10.1371/journal.pone.0143866 (PMC4666600; doi:10.1371/journal.pone.0143866)
Supplement: S9 Text — (DOCX) [file pone.0143866.s012.docx]

**S9 Text. Historical evidence and the 1400 clusters**

In 1400, the two northern clusters of higher grouped deaths were located in the historical centre of Dijon, the topography of which is well established. In the east, the limit of the cluster lies between the eastern side and the western side of *Fermerot* Street (see below). The two southern clusters of lower grouped deaths were identified by street names referring to the *Potet* and the *Crais* areas.

The low housing density in the southern part of Dijon is still apparent on the 1812 cadastral map. The *Potet* area was in direct contact with an estate belonging to the Saint-Etienne abbey, which was not yet sold for housing by the monks [Richard J. [Topography and history of Dijon: the "Old Castel"]. *Mémoires de la Commission des Antiquités de la Côte d'Or*. 1959-1962, 25, 253-282. French]. The *Crais* area was the last intramural part of the city where vineyards were present in the 15th century [Richard J. [Topographic history of Dijon: the city walls of Dijon from the 12th to the 16th century]. *ibid*, 1942-1946, 22, 320. French].

The location of the *Bourg* district suffers no ambiguity. Sources precisely mention that butchers households are in the area where they slaughter livestock, prepare and sell meet, between the western branch of the *Suzon* River and the remain of the ancient Dijon *castrum* fortification [Fyot E. [Dijon : its past recalled by its streets. New ed. Dijon: Damidot; 1980. p. 55-6] French]. The modern homolog of the area is still named *Bourg* Street and it is identified as *en la boucherie* ("in the butchery") in several *marcs* tax registers. A limited area including 105 households where most knife, sword and spur makers were settled was individualized around *Forges* Street. It was contiguous to the northern end of the Bourg district and crossed the *Suzon* River (Fig 1). Meat bakers were more numerous in a large cluster of 283 households. The corresponding area included in its eastern part the *Bourg* district as well as *Forges* Street and the street contiguous to its southern end (often identified as *Poulaillerie* by reference to hens). The 24 tanners and fishmongers were settled along the *Ouche* River, in the southern extramural part of Dijon (Fig 1) where their presence resulted in a cluster of 42 households.
