# Supplementary material for: Historical Epidemics Cartography Generated by Spatial Analysis: Mapping the Heterogeneity of Three Medieval "Plagues" in Dijon
Source: PLoS One. 2015 Dec 1;10(12):e0143866. doi: 10.1371/journal.pone.0143866 (PMC4666600; doi:10.1371/journal.pone.0143866)
Supplement: S10 Text — (DOCX) [file pone.0143866.s013.docx]

**S10 Text. Historical evidence and the 1428 clusters**

In the *Old Market* "stood second-hand clothing dealers, drapers, bakers" and "wheat was sold" [Colette E. [Fairs and markets of the old Dijon]. Dijon;1905, p 40-42. French]. The establishment of the *Champeaux* market place in 1326 is documented [Fyot E. *op. cit.* p. 488] and archeological remains still apparent on today's houses indicate its location. It was located at the south of *Archerie* Street, characterized by a mean tax level of 21 sols/taxpayer and a death rate of 23%. This restricted area of high mortality in *Archerie* Street would have escaped a death analysis based on parishes, as it was part of *Notre-Dame* parish where the general death rate was only 5%. This outlines the interest of cartographic analysis as a complement of analysis based on traditional subdivisions of urban space.

In 1428, the area of higher mortality lies in a part of the historic Dijon where street geography is unchanged and the correspondence between medieval and modern street names is known and sometimes preserved (for instance, *Archerie* Street is now *Auguste-Comte* Street, *Vannerie* Street is still *Vannerie* Street). This area included the northeastern part of *Saint-Michel* parish, location of the *Old Market*.
