# Supplementary material for: Historical Epidemics Cartography Generated by Spatial Analysis: Mapping the Heterogeneity of Three Medieval "Plagues" in Dijon
Source: PLoS One. 2015 Dec 1;10(12):e0143866. doi: 10.1371/journal.pone.0143866 (PMC4666600; doi:10.1371/journal.pone.0143866)
Supplement: S11 Text — (DOCX) [file pone.0143866.s014.docx]

**S11 Text. Historical evidence and 1438 and 1440 clusters**

In 1438, the area of higher mortality includes an extramural part and an intramural part of *Saint-Philibert* parish. In the former, fishmongers and tanners households are easy to spot along the *Ouche* River. In the latter, *Pont-Arnault* Street corresponds to the modern *Monge* Street (that preserves the meandering course of the now diverted *Suzon* river) and *Cloître* Street corresponds to the modern *Condorcet* Street (where still stands the historical house of the *Cîteaux* prior indicated in the *marcs* tax registers). The *Ouche* gate is the entry into the city for travellers coming from the South through the *Grand chemin de Beaune* ("large path from *Beaune*").

In 1440, the exact locations of the clusters of higher grouped deaths are not as firmly established because Dijon suburbs were destroyed at the beginning of the 16th century. They are situated in *Saint-Nicolas* suburb, on either side of the parish church and the neighbour *New Market*. The cluster of lower grouped deaths risk lies in the clearly defined intramural part.
